# Combination of dehydration and high light exposure effects on PSII photochemistry in three Antarctic moss species

**DOI:** 10.1093/aob/mcag207

**Published:** 2026-07-10

**Authors:** Davide Giordano, Syed Inzimam Ul Haq, Josef Hajek, Milos Bartak

**Affiliations:** Laboratory of Photosynthetic Processes, Department of Experimental Biology, Faculty of Science, Masaryk University, Brno 625 00, Czech Republic; Laboratory of Photosynthetic Processes, Department of Experimental Biology, Faculty of Science, Masaryk University, Brno 625 00, Czech Republic; Laboratory of Photosynthetic Processes, Department of Experimental Biology, Faculty of Science, Masaryk University, Brno 625 00, Czech Republic; Laboratory of Photosynthetic Processes, Department of Experimental Biology, Faculty of Science, Masaryk University, Brno 625 00, Czech Republic

**Keywords:** *Bryum pseudotriquetrum*, *Hypnum revolutum*, *Sanionia uncinata*, mosses, Antarctica, photosynthesis, dehydration, high light, photoinhibition, abiotic stress

## Abstract

**Background and Aims:**

Mosses stand out for their ability to sustain photosynthetic activity even when exposed to extreme stress. They have developed intricate protective strategies that safeguard photosynthesis from dehydration, excessive light and oxidative stress. While considerable progress has been made in understanding how dehydration and high light each affect PSII photochemistry, their combined influence on Antarctic mosses is still not well characterized. The present study examines how simultaneous dehydration and high light exposure influence PSII function in three Antarctic moss species: *Bryum pseudotriquetrum*, *Hypnum revolutum* and *Sanionia uncinata*.

**Methods:**

Chlorophyll fluorescence parameters, including *F*_V_/*F*_M_, *Φ*_PSII_, *R*_Fd_, NPQ, *q*_E_ and *q*_I_, were measured under controlled dehydration and high light treatments at the Mendel Polar Station on James Ross Island in the Antarctic peninsula; the obtained curves were analysed with generalized additive mixed models.

**Key Results:**

The results showed species-specific responses to the combined stress, displayed as a difference in curve shape, and a strong influence of high light on the desiccation curves’ shapes. *Bryum pseudotriquetrum* exhibited higher PSII performances under low light while *S. uncinata* showed a less rapid decline in photosynthetic efficiency under high light exposure and a lower NPQ decline in response to high light compared with the other species. It was observed that high light can strongly affect photosynthesis by enhancing the rate at which photosynthetic efficiency declines and at the same time induces a faster increase in photoinhibition-related quenching (i.e. *q*_I_).

**Conclusions:**

This study highlights interspecific differences among Antarctic mosses in their sensitivity to photoinhibition during thallus desiccation. Thus, light exposure during desiccation is an important factor to be considered in future ecophysiological studies. Finally, this study also emphasizes the need to examine both intra- and interspecific variability and the molecular and physiological mechanisms driving stress tolerance and adaptation in these species.

## INTRODUCTION

Photosynthesis regulation plays a central role in how plants adjust to changing and often harsh environmental conditions (Xia *et al.*, 2022). Among land plants, mosses stand out for their ability to sustain photosynthetic activity when exposed to extreme stress (Perera-Castro *et al.*, 2021*a*, 2022). Their transition from aquatic to terrestrial habitats has been tied to the emergence of intricate protective strategies that safeguard photosynthesis, particularly photosystem II (PSII), from dehydration, excessive light and oxidative stress (Yin *et al.*, 2023). In the harsh and highly variable conditions of polar ecosystems, Antarctic mosses serve as exceptional model organisms for exploring how photosynthesis copes with stress. These mosses face long periods of low temperatures, intense light and desiccation–rehydration cycles that, alone or in combination, can disrupt PSII photochemistry (Perera-Castro *et al.*, 2021*b*). Nevertheless, they maintain photosynthetic efficiency through diverse protective mechanisms, such as the temporary downregulation of PSII reaction centres, the engagement of the xanthophyll cycle and the accumulation of UV-absorbing pigments (Contreras and Zúñiga, 2025). These protective mechanisms help reduce photodamage by promoting thermal energy dissipation and supporting recovery once the tissues are rehydrated.

Among poikilohydric plants, dehydration represents one of the most severe environmental stressors, often causing a temporary shutdown of PSII reaction centres and a reduction in non-photochemical quenching (Bhatt *et al.*, 2024; Perera-Castro *et al.*, 2025). Research on Antarctic mosses reveals distinct tolerance capacities among species, with PSII quantum yield (*Φ*_PSII_) remaining largely stable until the relative water content (RWC) falls below ∼30 % (Orekhova *et al.*, 2022). When RWC drops below this threshold, sharp declines in *Φ*_PSII_ and steady-state fluorescence (*F*_S_) signal the onset of irreversible photoinhibition. High light stress can intensify negative effects of dehydration on PSII by decreasing its efficiency and overloading the photosynthetic electron transport chain, which in turn promotes the formation of reactive oxygen species (ROS) and decreases photochemical efficiency of PSII (Sachdev *et al.*, 2023; Yang *et al.*, 2023).

In Antarctic environments, episodes of intense irradiance may temporarily warm the moss canopy, enhancing enzymatic reactions but simultaneously increasing the risk of photoinhibition, especially when ambient temperatures remain low (Perera-Castro *et al.*, 2021*a*; Yin *et al.*, 2023). Typically, such events last tens of minutes and lead to a short-term photoinhibition of PSII, which is studied under simulated laboratory conditions in polar poikilohydric autotrophs, including mosses (Orekhova *et al.*, 2021), lichens (Haq *et al.*, 2024), biological crusts (Hejduková *et al.*, 2025) and terrestrial algae (Thangaraj, 2015; Permann *et al.*, 2022). Repetitive cycles of freezing and thawing may lead to reversible photoinhibition (Lovelock *et al.*, 1995).

In Antarctic field conditions, episodes of intense irradiance frequently coincide with desiccation events, particularly during freeze–thaw transitions and periods of intense midday irradiance, making their interaction an ecologically important determinant of moss resilience (Yin *et al.*, 2023). The majority of Antarctic mosses are considered resistant to short-term photoinhibition (Schroeter *et al.*, 2012) thanks to constitutive photoprotective mechanisms, including an effective xanthophyll cycle (García-Plazaola *et al.*, 2022), while chronic photoinhibition under combined low temperature and high light has also been documented (Perera-Castro *et al.*, 2021*b*).

Despite progress in understanding how dehydration and high light each affect PSII photochemistry in isolation, their combined influence on Antarctic mosses across the full desiccation range has not been characterized. The only prior study of combined desiccation and high light stress in mosses (Giudici, 2021) was limited to a single semi-hydrated state (87.5 % RWC), leaving the behaviour of PSII during progressive dehydration under sustained high light entirely unresolved. Moreover, while non-photochemical quenching (NPQ) has been used as a broad indicator of photoprotection in desiccation studies (Mishra *et al.*, 2024; Arslan *et al.*, 2025), its decomposition into the rapidly reversible energy-dependent component (*q*_E_) and the slowly reversible photoinhibitory component (*q*_I_) across the full desiccation curve has rarely been applied, and never under combined high light stress in Antarctic mosses. Resolving these gaps is important for understanding moss resilience under the increasingly frequent co-occurrence of desiccation and intense irradiance projected under Antarctic climate change scenarios.

The present study addresses this gap using three dominant Antarctic moss species from contrasting microhabitats on James Ross Island: *Bryum pseudotriquetrum* (open coastal oasis), *Hypnum revolutum* (persistently moist north-facing slopes) and *Sanionia uncinata* (topographically sheltered valleys). These species were selected since they represent the major bryophyte cover species in the northern Antarctic Peninsula yet differ in their exposure to irradiance and moisture availability, offering a natural gradient of potential photoprotective investment.

Chlorophyll fluorescence (ChlF) parameters related to PSII provide valuable insight into how plants respond to photoinhibition and balance photochemical efficiency with photoprotective energy dissipation under stressful conditions (Barták *et al.*, 2023). In this study we aimed to examine how simultaneous dehydration and high light exposure influence PSII function in three Antarctic moss species; we hypothesized that co-action of the two factors would accelerate photoinhibition-induced decline in PSII functioning when compared with desiccation alone (under low light). We also tested the hypothesis that co-action of the two factors would cause an earlier activation of protective mechanisms related to primary photosynthetic processes, in particular NPQ of absorbed light energy. By analysing chlorophyll fluorescence parameters related to NPQ, this work offers new insights into the strategies that allow Antarctic mosses to maintain a balance between photoprotection and photosynthetic activity under multiple environmental constraints. These findings may deepen our understanding of moss resilience mechanisms, shedding light on the ecological success of Antarctic bryophyte communities.

## MATERIALS AND METHODS

### Antarctic moss handling

We evaluated in this study three moss species: *Bryum pseudotriquetrum*, *Hypnum revolutum* and *Sanionia uncinata.* The samples were collected on James Ross Island in Antarctica in different sites where moss carpets were present (for coordinates and map, with pictures of the sites of collection of each species, see Fig. 2). When collected, the moss samples were brought to the Mendel Station facilities and hydrated with tap water (source of water: Bohemian stream, northern part of the Ulu Peninsula, James Ross Island) for one night before starting the experiment, to reach a RWC of 100 %. Two treatments, low light (LL) and high light (HL), were applied in desiccation experiments. For LL the moss samples were kept under natural light in the laboratory during the whole desiccation process (∼120 µmol m^−2^ s^−1^ of photosynthetically active radiation, PAR), and for HL the samples were kept under a strong light source (∼1500 µmol m^−2^ s^−1^of PAR) during the desiccation process; the light source for the HL treatment was an LED panel (TRON 250 × 250-L-830-830-CC-0, Tron, Brno, Czech Republic) (see Fig. 1 for the setup); light intensity was measured with an EMS 12 PAR sensor (light intensities were chosen to simulate intensities on overcast days or fully sunny days). A total of five replicates for each treatment were used, consisting of a moss sample with a 2-cm diameter. Moss samples were kept on ice for the whole duration of the experiment in order to keep a low temperature at the base of the sample; air temperature and humidity were monitored by using a Minikin THi (EMS Brno s.r.o.) data logger by placing it 2 cm from the samples. To calculate RWC, moss weight was recorded before each measurement (FWDD). The first weight — taken after one night of rehydration and removal of excess surface water — represented 100% RWC and was used as the fresh weight (FW). Dry weight (DW) was recorded after all measurements were completed, following overnight drying of the samples in an oven at 65°C. The RWC was calculated as follows:


RWC=(FWDD−DW)×100/(FW−DW).

**Fig. 1.**
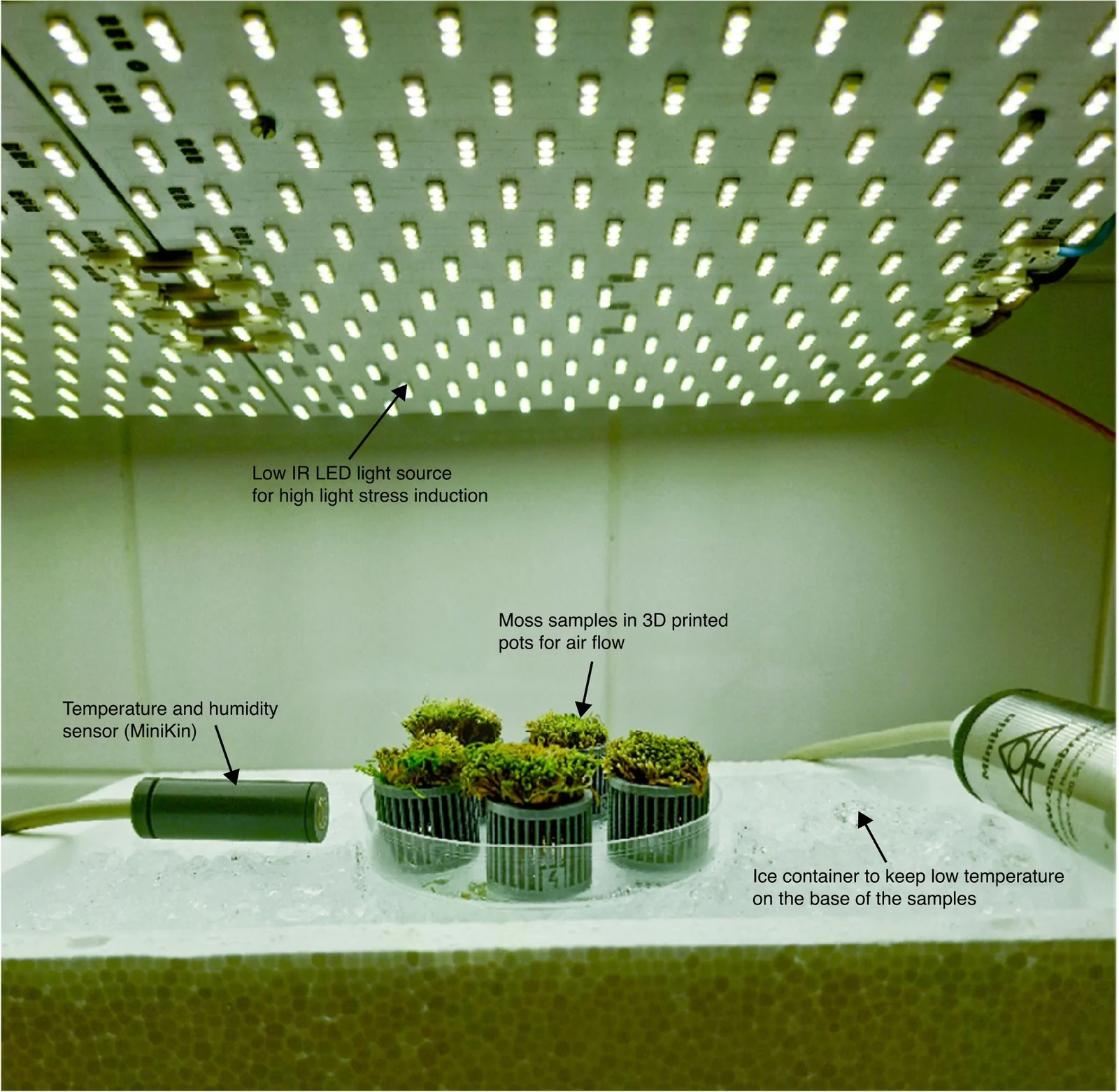
Representative picture of the experimental setup. The Minikin was used to measure the temperature and humidity of air near the samples. Moss samples were placed in 3-D printed pots that allow air flow through the moss sample. Samples that were not exposed to high light were placed under the laboratory’s natural light.

**Fig. 2.**
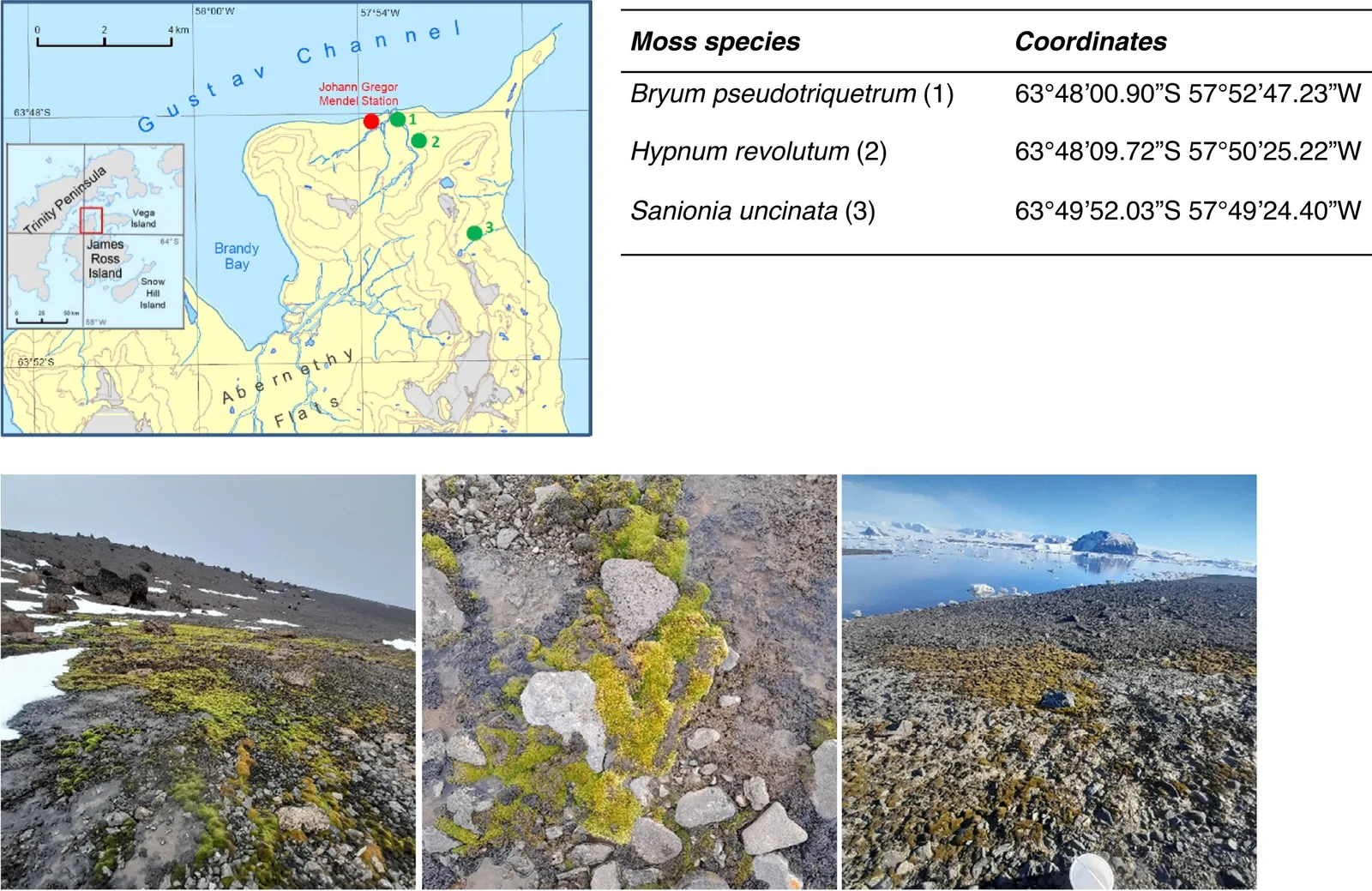
(Top) James Ross Island map with the sites of collection of the three species. On the right is a table with specific coordinates of the sites where the samples were collected. (Bottom) Sample collection sites at different Antarctic vegetation oases at James Ross Island. (Left) *Sanionia uncinata*; (centre) *Hypnum revolutum*; (right) *Bryum pseudotriquetrum*. Credits: Davide Giordano.

### Chlorophyll fluorescence

Chlorophyll fluorescence was measured during the whole desiccation process, with an interval of 20 min to 1 h between each measurement. To evaluate the effect of desiccation, with or without HL, we used the slow Kautsky kinetic (KK) technique. For this purpose, we used a Handy FluorCam (Photon Systems Instruments, Drásov, Czech Republic). Parameters derived from KK measurements are specified below. Mosses were dark-adapted for 10 min before starting the KK measurements. A single measurement started with a basal flash of light (0.5 µmol m^−2^ s^−1^of PAR) applied to dark-adapted leaves, inducing the basal fluorescence *F*_0_ while not activating photosynthesis; after 2 s a saturation pulse (3000 µmol m^−2^ s^−1^ of PAR, 0.8 s) induced the maximum chlorophyll fluorescence level (*F*_M_), followed by a 27-s dark period. Then the moss samples were exposed to a 300-s period of actinic light (AL) (60 µmol m^−2^ s^−1^ PAR), which activated the photosynthetic electron flow. After AL was switched on, the Kautsky effect (a rapid increase followed by a slow decline in fluorescence level) was recorded. After 300 s a steady state of ChlF was reached and a saturation pulse was applied to induce *F*_M_' (maximum chlorophyll fluorescence) in the light-adapted state. After switching off the actinic light, the background ChlF (*F*_0_') was recorded. A final saturation pulse was given then to induce *F*_M_' level in the dark and the KK end. The chlorophyll fluorescence parameters described in Table 1 were finally calculated by FluorCam software (Photon Systems Instruments, Drásov, Czech Republic) immediately after the measurement or during data elaboration using the script in the python programming language, where the data frame for final data analysis was created.

**Table 1.** ChlF parameters with description and equations.

ChlF parameter	Description	Equation
*F*_V_/*F*_M_	Potential quantum yield	(*F*_M_ − *F*_0_)/*F*_M_
*Φ*_PSII_	Effective quantum yield	(*F*_M_′ − *F*_0_)/*F*_M_′
NPQ	Non-photochemical quenching	(*F*_M_ − *F*_M_′)/F_M_′
*q*_I_	Photoinhibitory quenching	(*F*_M_ − *F*_M_′)/*F*_M_″
*q*_E_	Energy-dependent quenching	(*F*_M_″ − *F*_M_′)/*F*_M_′
*R*_Fd_	Chlorophyll fluorescence decrease ratio	(*F*_M_ − *F*_t_)/*F*_t_
NPQ (normalized)	Non-photochemical quenching (normalized on *F*_M_ control values)	*(*F*_M_ control − *F*_M_′)/*F*_M_′
*q*_I_ (normalized)	Photoinhibitory quenching (normalized on *F*_M_ control values)	*(*F*_M_ control − *F*_M_′)/*F*_M_″

^*^ *F*_M_ control is the maximum fluorescence in the dark-adapted state in the control group (i.e. samples at 100 % RWC).

### Data analysis

We modelled the response of chlorophyll fluorescence to changes in RWC using a generalized additive mixed model (GAMM) implemented in the mgcv package in R (v. 4.4.3, Wood, 2017). GAMMs are characterized by a sum of smooth functions that describe the relationship between predictors and the response variable, and they can capture complex, non-linear trends and interactions in the data while maintaining interpretability. GAMMs are particularly valuable when the underlying relationships are unknown or expected to vary smoothly, as they estimate these smooth functional forms directly from the data. An important advantage of this approach is its ability to represent complex patterns without imposing strong parametric assumptions, thus avoiding oversimplification. Compared with traditional non-linear regression, which requires specifying an explicit functional form (e.g. exponential, logistic or polynomial), GAMMs do not assume a particular mathematical shape for the relationship. Instead, they use data-driven smoothing techniques (such as splines) to flexibly adapt to the observed patterns. This makes GAMMs more versatile and robust when dealing with real-world data where the true form of the relationship is irregular or unknown. The model included treatment, species and their interaction as fixed parametric terms, and a smooth term for RWC that was allowed to vary by each species × treatment combination. The model formula was:


ChlF∼treatment×species+s(RWC,by=group)

where ChlF is the chlorophyll fluorescence parameter value, treatment is the light treatment (LL or HL), species is the moss species and group represents the interaction between species and treatment.

To account for non-independence among repeated measurements, replicate was included as a random intercept. Heterogeneous residual variances across species × treatment groups were modelled using a varIdent variance structure. The model was fitted by restricted maximum likelihood (REML). Model diagnostics (residual vs fitted plots, QQ plots and residual histograms) were inspected to verify normality and homoscedasticity of residuals (available in the Supplementary Data). Parametric coefficients that describe the linear effects of treatment, species and their interaction on each parameter, with *Bryum* under LL as the reference level, are shown in Table 2; approximate significances of smooth terms that describes the non-linear effect of RWC on each parameter within each group are shown as the edf (effective degree of freedom) in Table 3. The edf indicates the degree of non-linearity of the fitted spline (edf = 1, linear; higher values: increasingly non-linear). For NPQ_norm_ and *q*_Inorm_ (see Results section for further explanation), the inverse hyperbolic transformation (asinh) was used to improve the homoscedasticity. Model goodness of fit was evaluated using adjusted *R*^2^ (*R*^2^ adj), which represents the adjusted proportion of deviance explained by the model.

**Table 2.** Parametric coefficients describing the linear effects of treatment, species and their interaction on each ChlF parameter. The intercept (reference level) is set as *Bryum* under low light (LL). The adjusted *R*^2^ for each model is reported in the last row as a measure of goodness of fit.

	*F*_V_/*F*_M_	*Φ*_PSII_	*R*_Fd_	NPQ	*q*_I_	*q*_E_	NPQnorm (asinh)	qInorm (asinh)
Intercept	0.558***	0.429***	1.309***	0.330***	0.320***	0.016*	1.507***	1.506***
Treatment: HL	−0.116***	−0.121***	−0.515***	−0.076***	−0.082***	0.018	0.394***	0.373***
Species: *Hypnum*	−0.039**	−0.035**	−0.297***	0.008	−0.005	0.023***	0.104*	0.079
Species: *Sanionia*	−0.109***	−0.082***	−0.487***	−0.110***	−0.105***	−0.021*	0.178***	0.197***
Treatment × species: *Hypnum*	−0.037*	0.005	0.063	−0.074***	−0.061**	−0.061***	0.333***	0.870***
Treatment × species: *Sanionia*	0.064***	0.075***	0.342***	0.037 .	0.045*	−0.036*	0.080	0.114
*R* ^2^ (adj)	0.918	0.905	0.907	0.827	0.812	0.840	0.947	0.950

*** *P* < 0.001, ** *P* < 0.01, * *P* < 0.05, . *P* < 0.1.

**Table 3.** Approximate significance of smooth terms that describe the non-linear effect of RWC on each ChlF parameter within each group (species × treatment combination). Each value corresponds to effective degrees of freedom; edf = 1 indicates a linear relationship, while higher values reflect increasing non-linearity.

Group	*F*_V_/*F*_M_	*Φ*_PSII_	*R*_Fd_	NPQ	*q*_I_	*q*_E_	NPQ_norm_ (asinh)	*q*_Inorm_ (asinh)
*Bryum* LL	2.911***	3.043***	2.850***	2.429***	2.594***	2.089***	2.873***	2.846***
*Hypnum* LL	3.481***	3.594***	3.471***	3.494***	3.413***	3.636***	3.518***	3.513***
*Sanionia* LL	3.202***	3.447***	2.979***	2.101***	2.118***	1.839***	3.591***	3.522***
*Bryum* HL	2.529***	3.700***	2.765***	3.213***	3.259***	2.074***	3.420***	3.424***
*Hypnum* HL	3.484***	2.977***	3.560***	3.433***	3.438***	3.670***	3.395***	3.455***
*Sanionia* HL	3.562***	3.620***	3.306***	2.415***	2.117***	3.356***	3.747***	3.799***

*** *P* < 0.001, ** *P* < 0.01, * *P* < 0.05, . *P* < 0.1.

Differences between low LL and HL smooth functions for each species were calculated and plotted in R. Derivative functions of each smooth function were calculated in R to evaluate the slope values (plotted derivatives of smooth functions are shown in Supplementary Data Figs S1 and S2); differences between LL and HL derivative functions for each species were then plotted to evaluate the difference in slope between light treatments.

## RESULTS

### Temperature and relative humidity

The temperature and relative humidity recorded during the experiment are shown in Fig. 3. Under HL, air temperature ranged between 21 and 31 °C compared with 10–15 °C under LL, and relative humidity was 17–27 vs 40–50 % under LL. Moss samples were placed on ice to limit conductive heating from below; however, under HL treatment radiative heating from the LED elevated the air temperature above the sample. It is therefore important to note that the HL treatment represents a compound treatment in which elevated irradiance, higher air temperature and lower relative humidity co-occur; these conditions closely mirror natural Antarctic episodes of intense midday irradiance, where these three variables are coupled. Separating the independent contribution of irradiance from that of temperature and humidity would require a separate temperature-controlled HL experiment, including moss surface temperature control as well, which was beyond the scope of the present study. The potential contribution of temperature to the observed physiological responses is considered in the Discussion section.

**Fig. 3.**
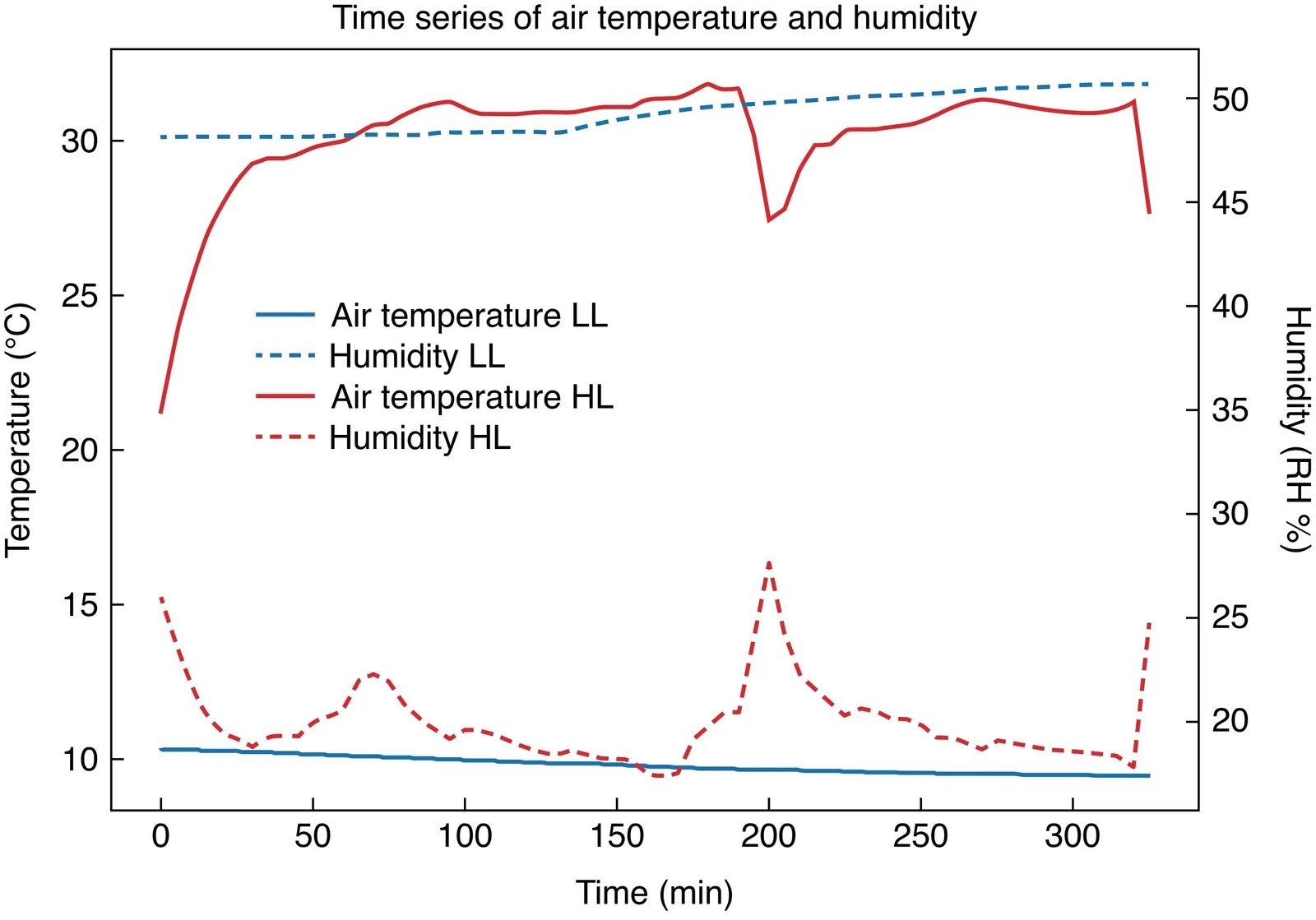
Time series of air temperature and relative humidity (RH%) in the laboratory where the experiment was conducted. Full lines indicate the air temperature, dashed lines indicate the relative humidity; LL is represented by blue colour and HL is red.

### RWC and high light-related changes in ChlF parameters

Figure 4 shows the curves of chlorophyll fluorescence parameters fitted through GAMMs. The GAMM revealed significant effects of treatment, species and their interaction on all the evaluated parameters (Table 2). The smooth functions describing the relationship between all ChlF parameters and RWC were highly significant for all species × treatment combinations (*P* < 0.001), with effective degrees of freedom between 1.7 and 3.8, indicating non-linear responses (Table 3). The parameters *F*_V_/*F*_M_, *Φ*_PSII_ and *R*_Fd_ describe the photosynthetic efficiency: *F*_V_/*F*_M_ is the potential quantum yield, *Φ*_PSII_ is the effective quantum yield, *R*_Fd_ is the chlorophyll fluorescence decrease ratio, a parameter more linked to the net photosynthesis (Genty *et al.*, 1989; Lichtenthaler *et al.*, 2005). They showed a sigmoid decline in response to desiccation; HL significantly increased the decline (*P* < 0.001), suggesting accelerated photoinhibition of PSII consistent with an additive effect of the HL stress with respect to the desiccation-induced stress. *R*_Fd_ showed high RWC-dependent variability; we attribute this added variability to the fact that *R*_Fd_ is calculated from the ratio of steady-state fluorescence and maximum fluorescence during the actinic light phase, making it sensitive not only to the photosynthetic state but also to small, transient changes in leaf orientation and self-shading within the moss sample during imaging (a source of optical noise that is largely irrelevant to *F*_V_/*F*_M_, measured in the dark-adapted state) but directly affects *R*_Fd_’s signal.

**Fig. 4.**
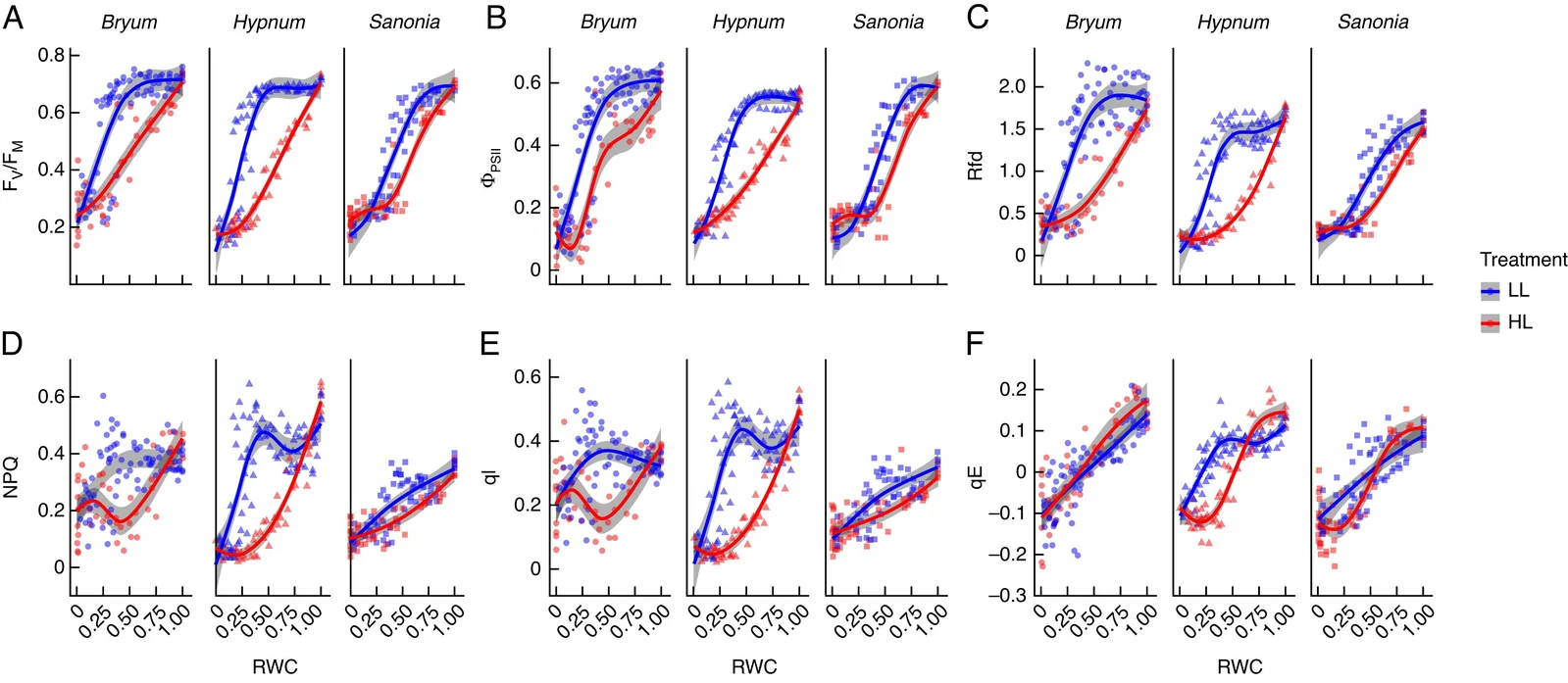
Desiccation curves of ChlF parameters (ChlF vs RWC). Blue represents low light (LL) desiccation curves and red represents high light (HL) desiccation curves. Grey areas represent 95 % confidence intervals. Species: *B. pseudotriquetrum*, circle; *H. revolutum*, triangle; *S. uncinata*, square. (A) *F*_V_/*F*_M_, (B) *Φ*_PSII_, (C) *R*_Fd_, (D) NPQ, (E) *q*_I_, (F) *q*_E_.

Figure 5 represents the difference between the smooth curves shown in Fig. 4 (LL − HL) for each species and parameter; LL values were significantly higher than HL between 10 and 80 % RWC, indicating a better state of the PSII functioning compared with HL, with no differences at the extremes of the curves, where the values were either optimal (at 100 % RWC, control conditions) or completely inhibited/stressed (0–10 % RWC). The only behaviour different from that of all the other parameters was for *q*_E_, where mosses exposed to HL showed higher values at higher RWC, to then drop faster than LL, changing the shape of the curve in a consistent way.

**Fig. 5.**
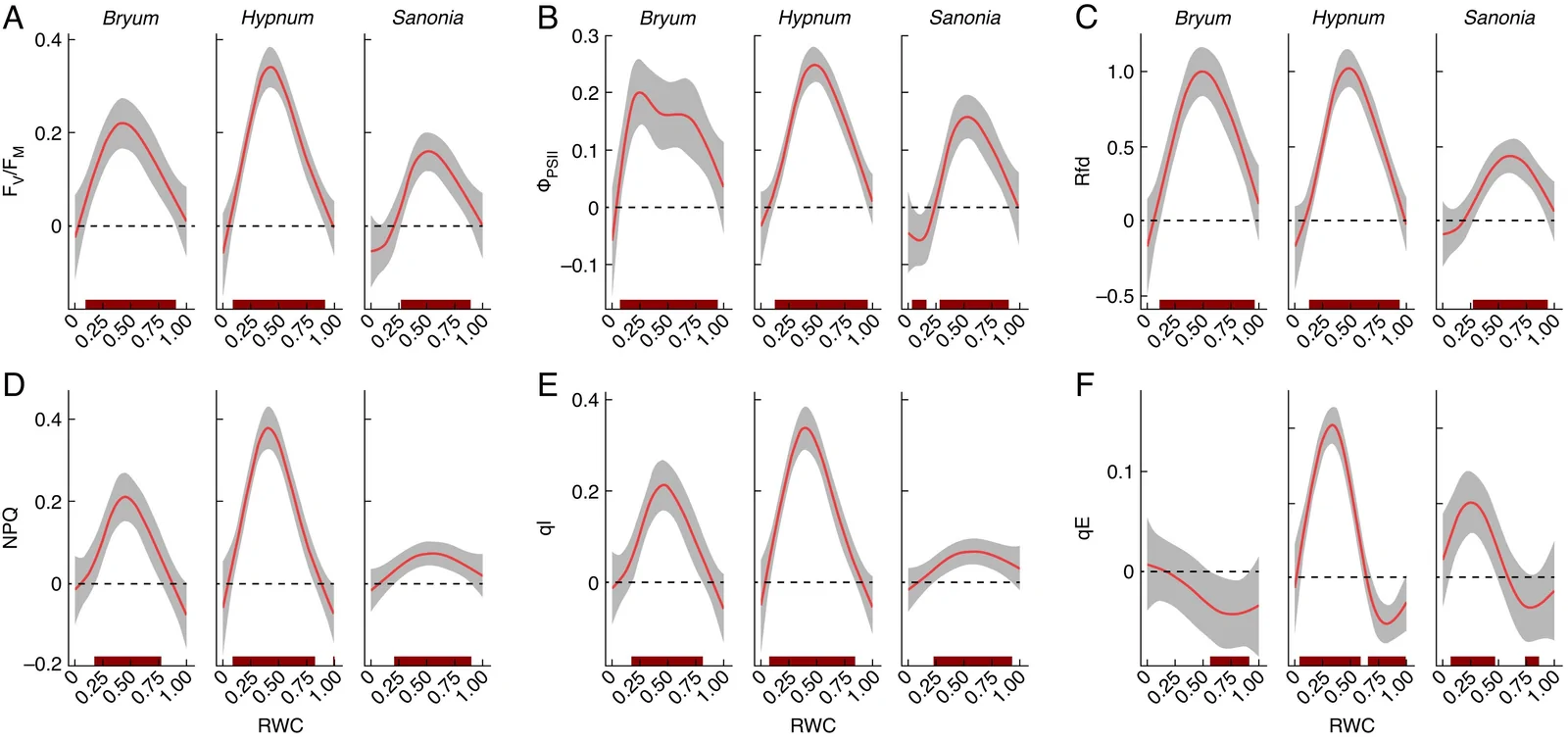
Differences between LL and HL smooth curves for each ChlF parameter, with 95 % confidence intervals. A difference was considered significant when the confidence interval did not intersect the zero line (dashed). Significant regions are highlighted by a red bar at the base of each panel. (A) *F*_V_/*F*_M_, (B) *Φ*_PSII_, (C) *R*_Fd_, (D) NPQ, (E) *q*_I_, (F) *q*_E_.

Figure 6 shows the difference between the derivatives of LL curves and HL curves; almost all the parameters followed a similar path, where HL induced a higher slope at RWC >50 %, while at RWC <25 % HL reached a plateau where the slope of LL curves was higher. Interestingly, this was different for *q*_E_, which saw a significant difference at ∼50 % RWC, with higher slopes of HL curves compared with LL curves.

**Fig. 6.**
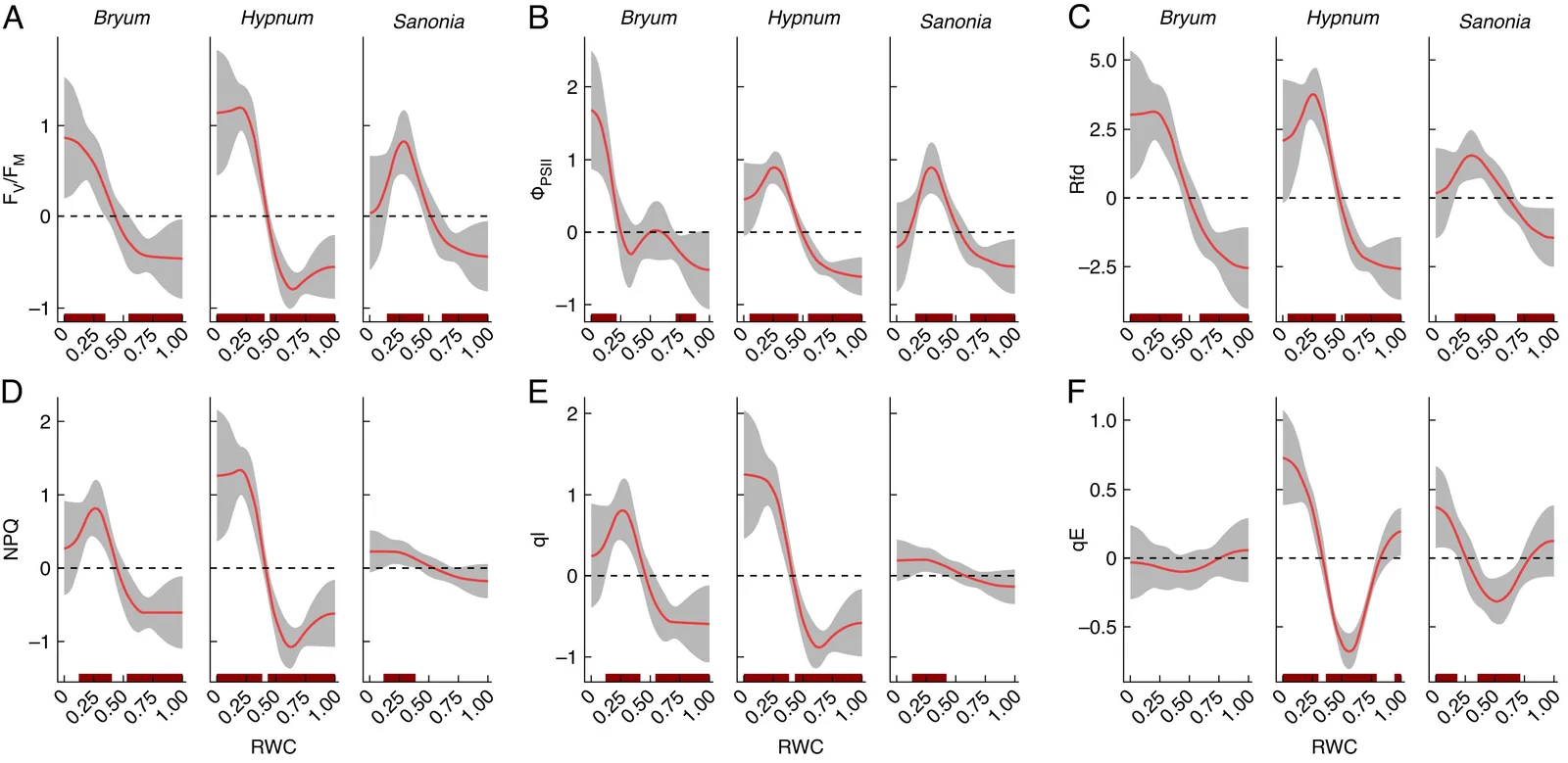
Differences between LL and HL derivative smooth curves for each ChlF parameter, with 95 % confidence intervals. A difference was considered significant when the confidence interval did not intersect the zero line (dashed). Significant regions are highlighted by a red bar at the base of each panel. (A) *F*_V_/*F*_M_, (B) *Φ*_PSII_, (C) *R*_Fd_, (D) NPQ, (E) *q*_I_, (F) *q*_E_.

The parameters *q*_I_ and *q*_E_ are components of NPQ, the fraction of light energy that is dissipated as heat: *q*_I_ is photoinhibitory quenching, a slowly reversible quenching more related to the photoinhibition of PSII and the inactivation of the D1 protein; *q*_E_ is energy-dependent quenching, a rapidly reversible and *Δ*pH-dependent component that is related to the xanthophyll cycle. NPQ, *q*_I_ and *q*_E_ exhibited a high degree of variability and non-linearity (edf >2); although their values were not the same, NPQ and *q*_I_ followed a mirror-like pattern, suggesting that the latter composed a major part of NPQ compared with *q*_E_. All of them, moreover, declined in response to desiccation, and HL determined a more rapid decline (*P* < 0.001). NPQ and *q*_I_ followed an initial increase in response to desiccation, between 75 and 25 %, to then decline, more or less sharply according to the species (*H. revolutum* had a sharper decline than the other two species); HL determined a sharper decline from the beginning of the curve, which did not show the initial increase in NPQ and *q*_I_ observed under natural light conditions. Parameter *q*_E_ followed a different pattern: it started with higher values under HL compared with LL; however, HL caused a steeper decline in the intermediate RWC range (75–25 %), reflecting a shift from reversible energy-dependent quenching towards photoinhibitory quenching as desiccation advanced.

Standard NPQ and *q*_I_ calculations use the instantaneous *F*_M_ measured at each time point as the denominator. During severe desiccation, *F*_M_ itself declines substantially due to water loss, which reduces ChlF emission, meaning that conventional NPQ values can appear to decrease under a certain RWC range. To investigate the ratio of thermal dissipation relative to a fully functional PSII, NPQ and *q*_I_ were additionally normalized to the *F*_M_ value recorded at full hydration in control samples (NPQ_norm_, *q*_Inorm_; see Table 1 for equations). Under this normalization, both NPQ_norm_ and *q*_Inorm_ rose progressively with desiccation (Fig. 7A), a pattern that is the inverse of standard NPQ and *q*_I_, and that more accurately reflects the actual increase in energy dissipation per unit PSII capacity as stress advances. High light induced a faster and larger rise in both normalized parameters, with values remaining elevated even at very low RWC (Fig. 7B, C). This normalization also substantially improved model fit (*R*^2^ adj from 0.82 to 0.95 for NPQnorm; Table 2). A further improvement was achieved by applying an inverse hyperbolic sine (asinh) transformation to stabilize heteroscedasticity across the wide dynamic range of these parameters (*R*^2^ adj 0.90 untransformed vs 0.95 transformed, data from untransformed not shown), producing a more reliable model; the inverse hyperbolic sine (asinh) transformation is particularly effective when the range of the data (minimum and maximum) is wider, such as the case of NPQ_norm_ and *q*_Inorm_; with parameters like *F*_V_/*F*_M_ that have a range of 0–0.85 this transformation would not have the same effect but a negligible one.

**Fig. 7.**
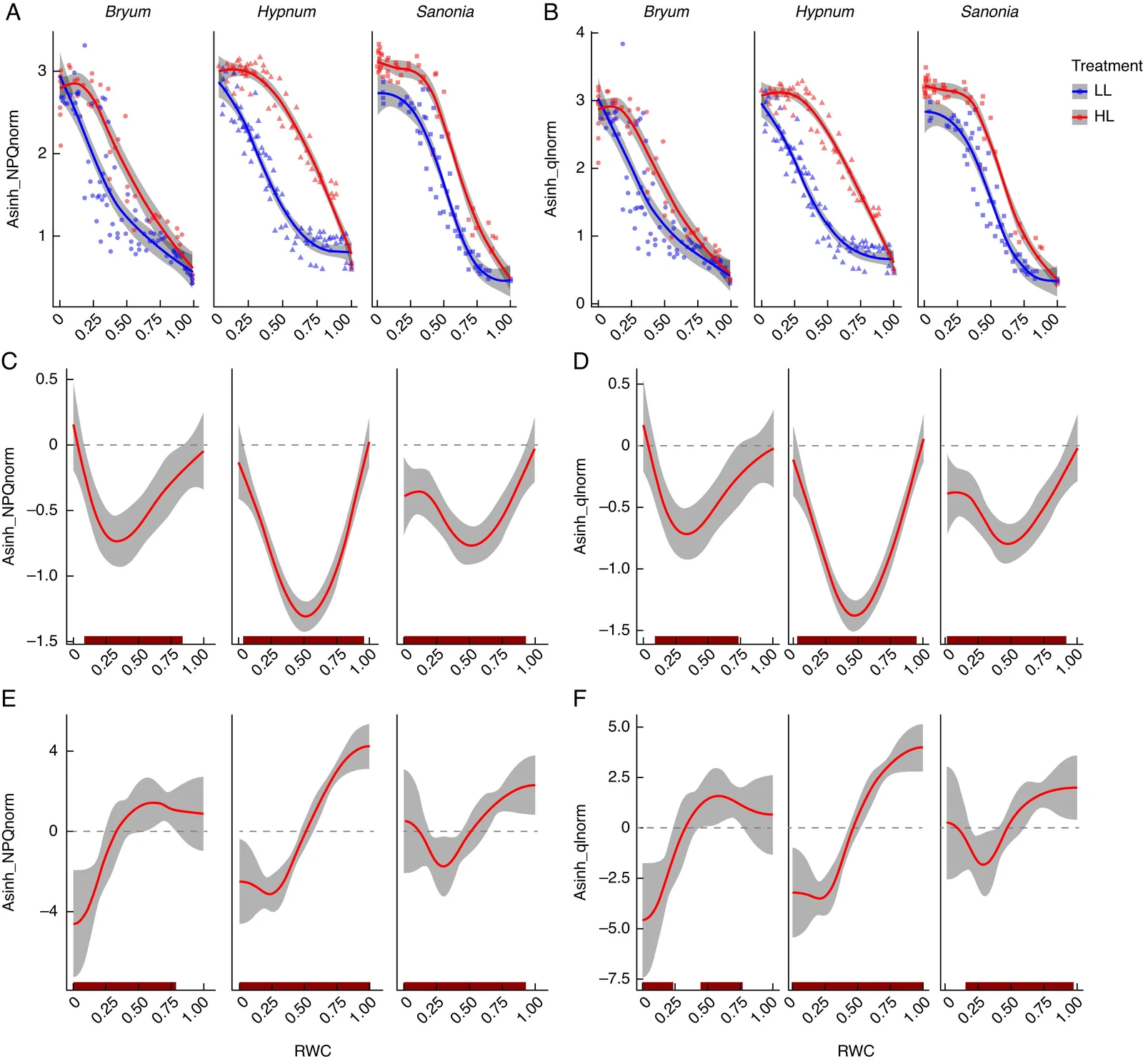
Desiccation curves (ChlF vs RWC) of normalized NPQ and *q*_I_ transformed using the inverse hyperbolic transformation (A, B); differences between LL and HL smooth curves, with 95 % confidence intervals (C, D); difference between LL and HL derivative smooth curves with 95 % confidence interval (E, F).

### Species-specific differences

Under LL, *H. revolutum* and *S. uncinata* exhibited lower *F*_V_/*F*_M_ and *Φ*_PSII_ than *B. pseudotriquetrum* (*P* < 0.01). High light significantly reduced both the parameters in *B. pseudotriquetrum* (*P* < 0.001) and *H. revolutum*, whereas *S. uncinata* showed a less pronounced HL-dependent decline. *Hypnum revolutum* showed higher *R*_Fd_ values than *B. pseudotriquetrum* and *S. uncinata*, whose curve shapes were similar, but also showed a stronger effect of HL on the decline of *R*_Fd_ values across the RWC; moreover, *H. revolutum* showed what is called a supersaturation effect, as seen also in the *F*_V_/*F*_M_ curve, i.e. a slight decline in the 75–100 % range of RWC.

All species tended to have a lower NPQ and *q*_I_ under HL, but the magnitude differed. *Hypnum* dropped more strongly than *Bryum* under HL, while *Sanionia* resisted the decrease. Under LL, *Sanionia* already had a lower NPQ and *q*_I_ than the other two species.

As for the normalized NPQ and *q*_I_, *H. revolutum* under LL showed no difference from the reference *Bryum* under LL, while under HL NPQ increased more in *H. revolutum* than *B. pseudotriquetrum*, and the increases in NPQ_norm_ and *q*_Inorm_ in *S. uncinata* under HL were not significant compared with the reference *B. pseudotriquetrum*. Finally, the decline observed in *q*_E_ was most pronounced in *H. revolutum* and *S. uncinata*, both of which showed significant negative treatment × species interactions, indicating a stronger HL-induced suppression of *q*_E_ relative to *B. pseudotriquetrum*. *Hypnum revolutum* displayed the most non-linear relationship under both treatments (edf >3.5), while *S. uncinata* showed the most linear response under LL.

## DISCUSSION

### Photoinhibition under combined desiccation and high light exposure

This study provides the first characterization of PSII photochemical responses across the full desiccation range under sustained high irradiance in Antarctic mosses, extending previous single-RWC-point assessments (Giudici, 2021) and single-factor desiccation studies (Orekhova *et al.*, 2022; Arslan *et al.*, 2025). The results support both hypotheses: combined desiccation and HL (1) accelerated PSII decline and (2) triggered an earlier shift from reversible to photoinhibitory quenching, with significant species-specific differences in the magnitude of both responses. The threshold RWC at which full PSII inhibition occurred shifted from 0–10 % under LL to 0–25 % under HL across all three species, consistent with ROS-mediated oxidative damage to PSII reaction centres occurring before water deficit alone reaches a structurally damaging level (Bhatt *et al.*, 2023). Desiccation suppresses moss photosynthesis through progressive structural and metabolic disruption: initial turgor loss limits CO_2_ assimilation and growth, while more severe dehydration destabilizes thylakoid membranes and pigment–protein complexes, ultimately collapsing the electron transport (Black and Pritchard, 2002; Proctor and Tuba, 2002; Challabathula *et al.*, 2016). Under HL, excess excitation energy is expected to drive ROS accumulation, which accelerates D1 protein inactivation and impairs the repair cycle (Murata *et al.*, 2007; Kirchhoff 2014; Sharma *et al.*, 2023), raising the RWC level at which PSII centres are effectively damaged. This ROS-driven mechanism was compounded in our experiment by the higher air temperature and lower relative humidity under HL (21–31 °C and 17–27 % RH vs 10–15 °C and 40–50 % RH under LL), which independently accelerate desiccation rate and may additionally affect PSII membrane fluidity and NPQ activation. While the coupled nature of the HL treatment precludes full attribution of the observed responses to irradiance alone, it more faithfully reproduces the stress conditions that Antarctic mosses experience during natural high-irradiance events, where intense solar radiation, elevated canopy temperature and high evaporative demand may co-occur routinely (Yin *et al.*, 2023).

The sigmoid kinetics of *F*_V_/*F*_M_, *Φ*_PSII_ and *R*_Fd_ decline are consistent with a threshold-dependent mechanism, as observed in other studies (Hájek *et al.*, 2022; Andrzejowska *et al.*, 2024): a slow initial phase during which photoprotective mechanisms buffer PSII efficiency, followed by a rapid collapse once these mechanisms are exhausted (in our case below 50 % RWC).

The desiccation-induced decline in *F*_V_/*F*_M_ can reflect a decline in *F*_M_, a rise in *F*_0_, or both. In our experiment, both *F*_M_ and *F*_0_ declined progressively with desiccation (Fig. 8, Supplementary Data S2, Supplementary Data Tables S1 and S2), but *F*_M_ declined faster than *F*_0_, and this asymmetry is what drove the decline in *F*_V_/*F*_M_ below ∼30 % RWC. This merges with the NPQ and *q*_I_ pattern and is consistent with the formation of quenching centres in PSII reaction centres during desiccation, a mechanism described in detail in other poikilohydric mosses (Heber *et al.*, 2006; Heber, 2008) in which reductive charge stabilization in the reaction centre is replaced by non-radiative energy dissipation at the QA level (see next section, about NPQ).

**Fig. 8.**
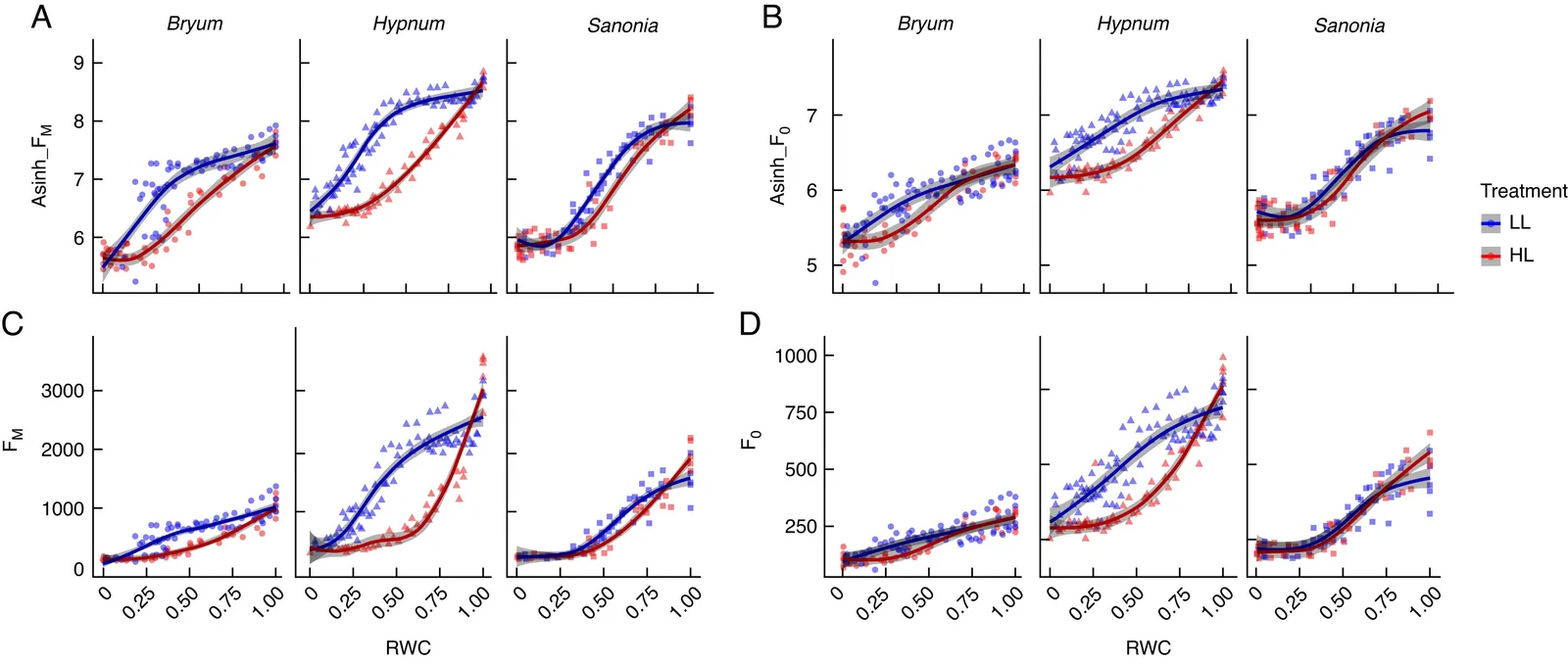
desiccation curves of ChlF parameters (ChlF vs RWC). Blue colour indicates LL desiccation curves and red indicates HL desiccation curves. Grey areas represent 95 % confidence intervals. Species: *B. pseudotriquetrum*, circle; *H. revolutum*, triangle; *S. uncinata*, square. (A) Transformed *F*_M_ (asinh_F_M_), (B) transformed *F*_0_ (asinh_F_0_), (C) *F*_M_, (D) *F*_0_.

### NPQ dynamics reveal a shift from photoprotection to photodamage

A key novelty of this study is the comprehensive decomposition of NPQ into its components – *q*_E_ and *q*_I_ – across the full desiccation curve and under both light treatments. While NPQ has been widely used as an indicator of the status of protective mechanism activation in response to desiccation (Mishra *et al.*, 2024; Arslan *et al.*, 2025), this level of NPQ resolution, which evaluates in a deeper way the kinetics of combined desiccation and HL, has been rarely applied in desiccation studies.

Under LL, NPQ and *q*_I_ follow a characteristic rise between ∼75 and ∼25 % RWC before declining sharply. This pattern reflects an initial activation of photoprotective dissipation in response to the declining water content, followed by a desiccation damage-related quenching decline (Proctor and Tuba, 2002). Under HL, this initial rise is abolished. A decline in NPQ under severe desiccation stress has been reported in other poikilohydric organisms (Arslan *et al*., 2025); what we see from our results indicates that HL determines greater oxidative damage and does not allow the photosynthetic apparatus to cope with the desiccation-related stress in the most efficient way.

Our experiment, focused on PSII functioning and NPQ activation during desiccation under LL and HL treatments, mimicked ecophysiological behaviour in the field. Our experimental moss species were collected in open habitats, presumably being acclimated to HL. Thus, the NPQ activation during desiccation found for the three experimental species and the underlying mechanisms should be attributed to those reported for sun-adapted mosses. Yamakawa and Itoh (2013) first suggested the activation of specific NPQ mechanisms in response to dryness, designated d-NPQ; then, Yamakawa *et al.* (2018) suggested two energy dissipation mechanisms involved in the excess excitation energy dissipation from PSII for sun- and shade-adapted mosses, respectively. In the first one (A-type) quenching is characterized by a desiccation-induced quencher inside the PSII core. This type is seen in sun-adapted mosses, while B-type quenching, typical of shade-adapted mosses, works mainly due to a decrease in the excitation energy from PSII probably situated in a light-harvesting complex (LHC) or PSII peripheral. The above-mentioned formation of quenching centres during moss desiccation is consistent with the concept of Heber *et al.* (2006), who reported highly effective quenching centres of PSII induced by desiccation (Heber, 2008); this is consistent with the initial rise of NPQ and *q*_I_, followed by a decline, observed in our study.

The *q*_E_ pattern reinforces this interpretation. Under both treatments, *q*_E_ is initially elevated, reflecting fast-activated reversible photoprotection, associated with zeaxanthin formation, consistent with the high constitutive xanthophyll pool reported in Antarctic mosses (García-Plazaola *et al.*, 2022) and the LHC-localized *q*_E_ mechanism characterized in *Physcomitrella patens* (Pinnola *et al.*, 2013). To cope with a variety of stressors inhibiting photosynthetic activity, Antarctic mosses possess, apart from zeaxanthin associated with *q*_E_, several constitutive antioxidants effective in photoprotection, such as phenolics (Rozema *et al.*, 2001), flavonoids (Bhattarai *et al.*, 2008) and UV screens/antioxidants (Waterman *et al.*, 2017). However, under HL, *q*_E_ declines more steeply in the 75–25 % RWC range, while *q*_Inorm_ continues to rise; this reciprocal dynamic represents a mechanistic shift from reversible, energy-dependent quenching towards the slow photoinhibitory quenching, with the crossover occurring at higher RWC under HL than under LL.

A critical methodological issue in desiccation fluorescence studies is that *F*_M_ itself declines with dehydration, causing conventional NPQ to be underestimated as stress intensifies. By normalizing NPQ and *q*_I_ to the control *F*_M_ value at full hydration (yielding NPQ_norm_ and *q*_Inorm_), we recovered a parameter that reflects the total energy dissipated as heat relative to a fully functional PSII pool, including the contribution from photodamaged centres. Under this normalization, both parameters rise with desiccation, and HL amplifies this rise (a pattern that is obscured in conventional NPQ plots where severe stress appears to reduce quenching simply because *F*_M_ collapses). This normalization also substantially improved model performance (*R*^2^ from 0.82 to 0.95) and, combined with an inverse hyperbolic transformation to stabilize heteroscedasticity, produced further improvement, suggesting a more stable NPQ among the replicates; the model improvement reflects a genuine gain in biological signal-to-noise, not a modelling convenience: the normalized parameter is simply less contaminated by the fluorescence-optical collapse that obscures quenching dynamics at low RWC. We suggest that *F*_M_-normalized NPQ should be adopted as a standard reporting practice in desiccation fluorescence studies, particularly where the full desiccation range is sampled.

### Interspecific differences reflect niche-specific photoprotective strategies

The three species differed in a significant way. *Bryum pseudotriquetrum* maintained the highest *F*_V_/*F*_M_ and *Φ*_PSII_ under LL, consistent with its documented desiccation tolerance (Robinson *et al.*, 2000) and recent evidence that stress-responsive lipid metabolism and oil body formation underpins its cellular protection during dehydration (Otani *et al.*, 2024). *Sanionia uncinata* showed the greatest resistance to HL-induced ChlF decline, suggesting a constitutive investment in photoprotective mechanisms such as pigments or antioxidant capacity (phenolics, flavonoids, UV screens; Waterman *et al.*, 2017) that buffers PSII against oxidative stress independently of water status. *Hypnum revolutum*, by contrast, displayed the steepest ChlF collapse under HL, and a more pronounced supersaturation effect at near-full hydration. Differences in primary photosynthetic responses under combined desiccation and HL may be niche-specific as well. *Bryum pseudotriquetrum* was sampled in the area close to the J.G. Mendel Polar Station (Area No. 1 in Barták *et al.*, 2015), an open coastal vegetation oasis at low elevation; *H. revolutum* was collected from the meltwater seepage on the north-facing foothill of Berry Hill mesa (Komárek's slopes, Area No. 2 in Barták *et al.*, 2015; Skácelová *et al.*, 2015), a persistently moist habitat continuously supplied by snowfield and permafrost meltwater; *S. uncinata* was sampled from the informally called Paradise Valley (Area No. 5 in Barták *et al.*, 2015), a topographically sheltered shallow valley where large boulders promote snow accumulation and consequent slow melting due to the south-eastern exposure of the valley. Although dedicated studies of niche-specific microclimatic conditions and their relationship to moss photosynthetic behaviour remain limited, some interpretation is possible from the existing literature (Láska *et al.*, 2011; Barták *et al.*, 2015; Skácelová *et al.*, 2015). The coastal area where *B. pseudotriquetrum* was collected is subject to the full range of coastal irradiance and wind exposure typical of the coastal vegetation oases. The high PSII performance of *B. pseudotriquetrum* under LL and its relatively steep decline under HL may reflect an ecotype acclimated to moderate, variable irradiance at an open coastal site, rather than constitutive HL resistance*. Hypnum revolutum* was collected from a north-facing slope with higher direct irradiance exposure compared with south-facing sites, and the continuous moisture supply from multiple streams creates conditions where desiccation is less frequent compared with open coastal areas. Moisture availability seems to be a driving factor at this site since it remains wet even if surrounding habitats become dry (M. Barták, pers. comm.). The particularly steep collapse of ChlF parameters in *H. revolutum* under HL treatment may therefore reflect a lower constitutive investment in HL photoprotection in a species growing in a relatively buffered, moisture-rich microhabitat. *Sanionia uncinata* displayed the greatest resistance to HL-induced ChlF decline, suggesting a generally high, constitutive photoprotective capacity, since the species is reported from sunlit plots not only from James Ross Island but also from many other islands west of the Antarctic Peninsula. Future work combining moss canopy microclimate measurements at these specific sites with molecular characterization of photoprotective pathways will be necessary to move beyond these interpretations and establish a link between niche-specific conditions and photosynthetic stress tolerance in Antarctic mosses.

As ChlF proved to be a great tool for fast detection of differences, further studies (such as genetics and metabolomics) would surely improve our understanding of the mechanistic aspects of biodiversity in moss stress tolerance, especially in less studied species such as *H. revolutum*.

The differences found in this study underscore that one-point desiccation tolerance metrics are insufficient to predict species responses under realistic field conditions, where high irradiance and dehydration co-occur routinely. The finding that combined desiccation and HL produces qualitatively different, and not just quantitatively enhanced, photosynthetic responses has direct consequences for how Antarctic moss physiology is modelled in the context of climate change. Projections based on single-factor desiccation experiments can significantly underestimate PSII state under fluctuating light. Microclimatic heterogeneity within a single moss carpet can expose adjacent shoots to different irradiance and moisture regimes (Randall, 2022; Yin *et al.*, 2023), and our data suggest the photosynthetic consequences of such variation are complex, non-linear and species-dependent.

Some limitations of the present study should be acknowledged. First, as discussed above, the HL treatment affected air temperature and relative humidity, precluding clean attribution of responses to irradiance alone; a temperature-controlled HL experiment would be needed to isolate the effect of HL. Second, recovery measurements following rehydration or HL were absent, following the open question of whether the accelerated photoinhibition observed under HL represents reversible or irreversible damage, an important distinction for understanding species-specific moss resilience under field conditions. Third, the absence of biochemical and molecular analysis prevents direct mechanistic confirmation of the pathways inferred from ChlF results. Fourth, with five replicates per treatment and species, statistical power for detecting subtle interspecific differences in some parameters (particularly *q*_E_) is limited. These limitations identify priority directions for follow-up work: temperature-controlled stress experiments, recovery kinetics and combined fluorescence–pigment–proteomics approaches would substantially strengthen mechanistic conclusions.

From a methodological perspective, the use of GAMMs proved to represent a good, more flexible, alternative to the linear and polynomial regression approaches predominant in the desiccation fluorescence literature. The flexibility of the model allowed the data to reveal important insights into stress physiology without constraining them to a predetermined functional form.

### Conclusions

Changes in water availability affecting hydration/desiccation cycles of mosses in Antarctica, together with contrasting light intensity (high on fully sunny days and low on overcast days), are driving factors for *in situ* photosynthetic performance of Antarctic mosses. This study addresses this co-action, confirming the two hypotheses that exposure to HL significantly accelerates PSII inactivation during desiccation and triggers photoprotective mechanisms across all three experimental species.

The evaluation of several ChlF parameters together with the decomposition of NPQ into *q*_E_ and *q*_I_ across the full desiccation curve, the normalization of these parameters to control *F*_M_ values and the use of GAMMs represents a methodological contribution applicable to future desiccation fluorescence studies. The three species responded to combined stress in qualitatively and quantitatively distinct ways: *B. pseudotriquetrum* showed the highest photosynthetic efficiency under LL while *S. uncinata* exhibited greatest resistance to HL-induced decline.

Future studies should incorporate fluctuating light regimes that more closely reflect natural field conditions, evaluating PSII recovery following rehydration and relaxation from photoinhibition, and investigating the underlying molecular and physiological mechanisms of stress tolerance (particularly in less-studied species such as *H. revolutum*). Finally, this study highlights the importance of examining intraspecific variability alongside interspecific variability, as both levels of variation are crucial for understanding stress tolerance and adaptation in Antarctic mosses. Such investigations will be important for a more comprehensive understanding of Antarctic moss biodiversity and its vulnerability to a rapidly changing climate in the majority of coastal moss-dominated vegetation oases in Antarctica.

## Supplementary Material

mcag207_Supplementary_Data

## AI ASSISTANCE

Grammarly software was used for language and grammar checks
