# Supplementary material for: Combination of dehydration and high light exposure effects on PSII photochemistry in three Antarctic moss species
Source: Ann Bot. 2026 Jul 10;138(5):1943–55. doi: 10.1093/aob/mcag207 (PMC13715043; doi:10.1093/aob/mcag207)
Supplement: Supplementary file 1 [file mcag207_supplementary_data.zip › diagnostic_plots.pdf]

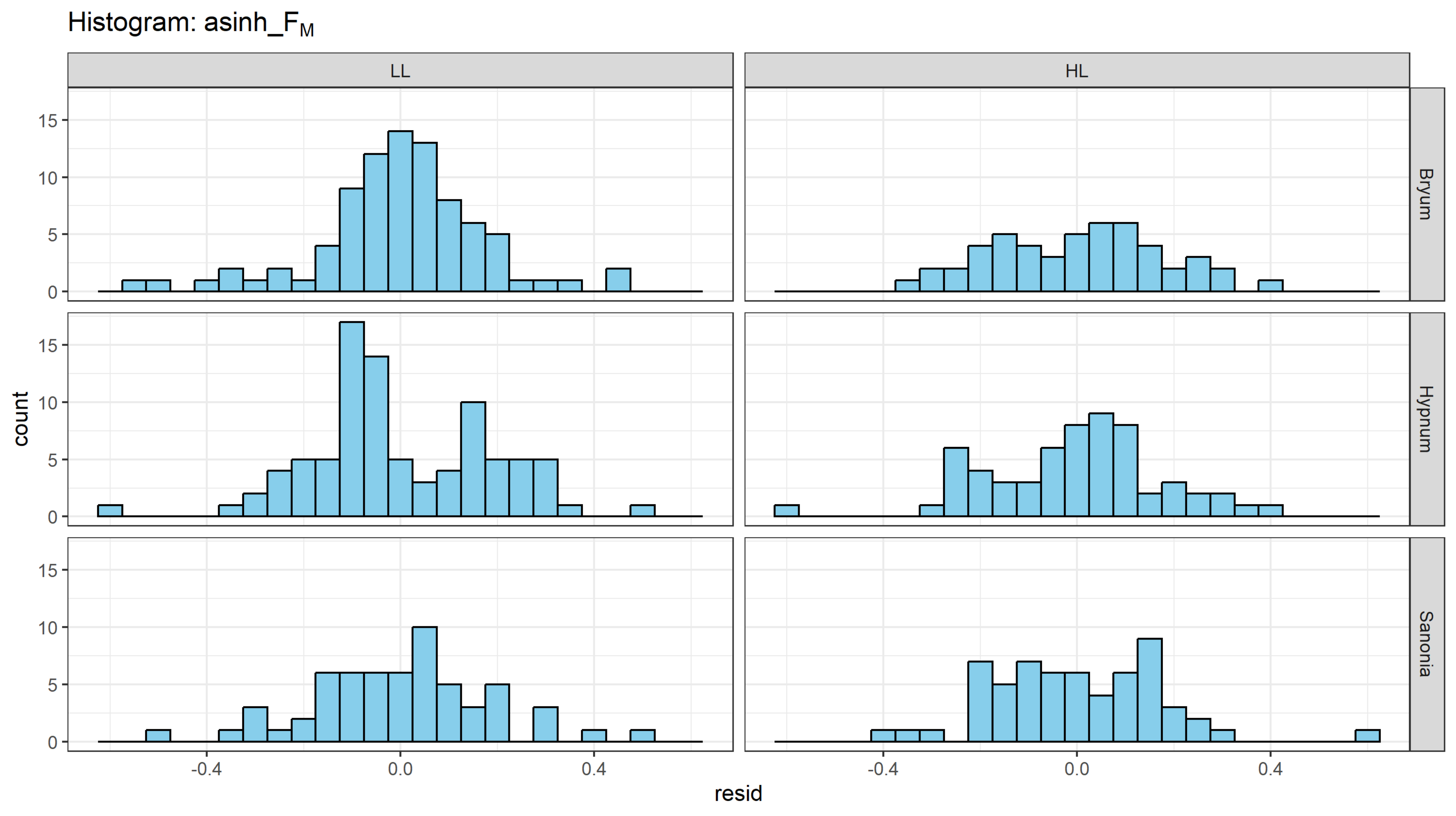

Histogram: asinh_F_M
LL
HL
Bryum
Hypnum
Sanonia
count
resid
15
10
5
0
-0.4
0.0
0.4

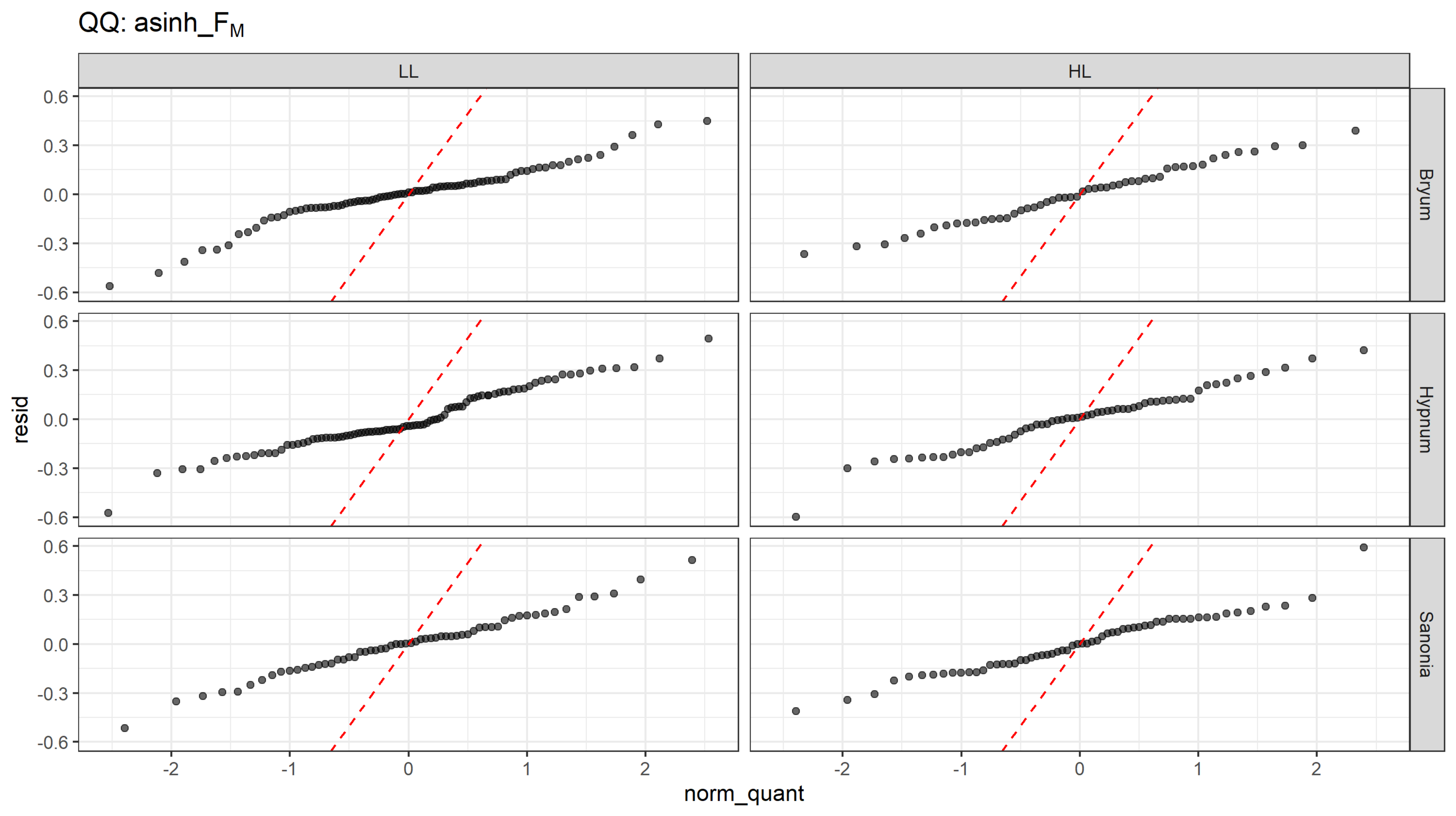
QQ: asinh_F_M
LL
HL
Bryum
Hypnum
Sanonia
resid
norm_quant
0.6
0.3
0.0
-0.3
-0.6
-2
-1
0
1
2

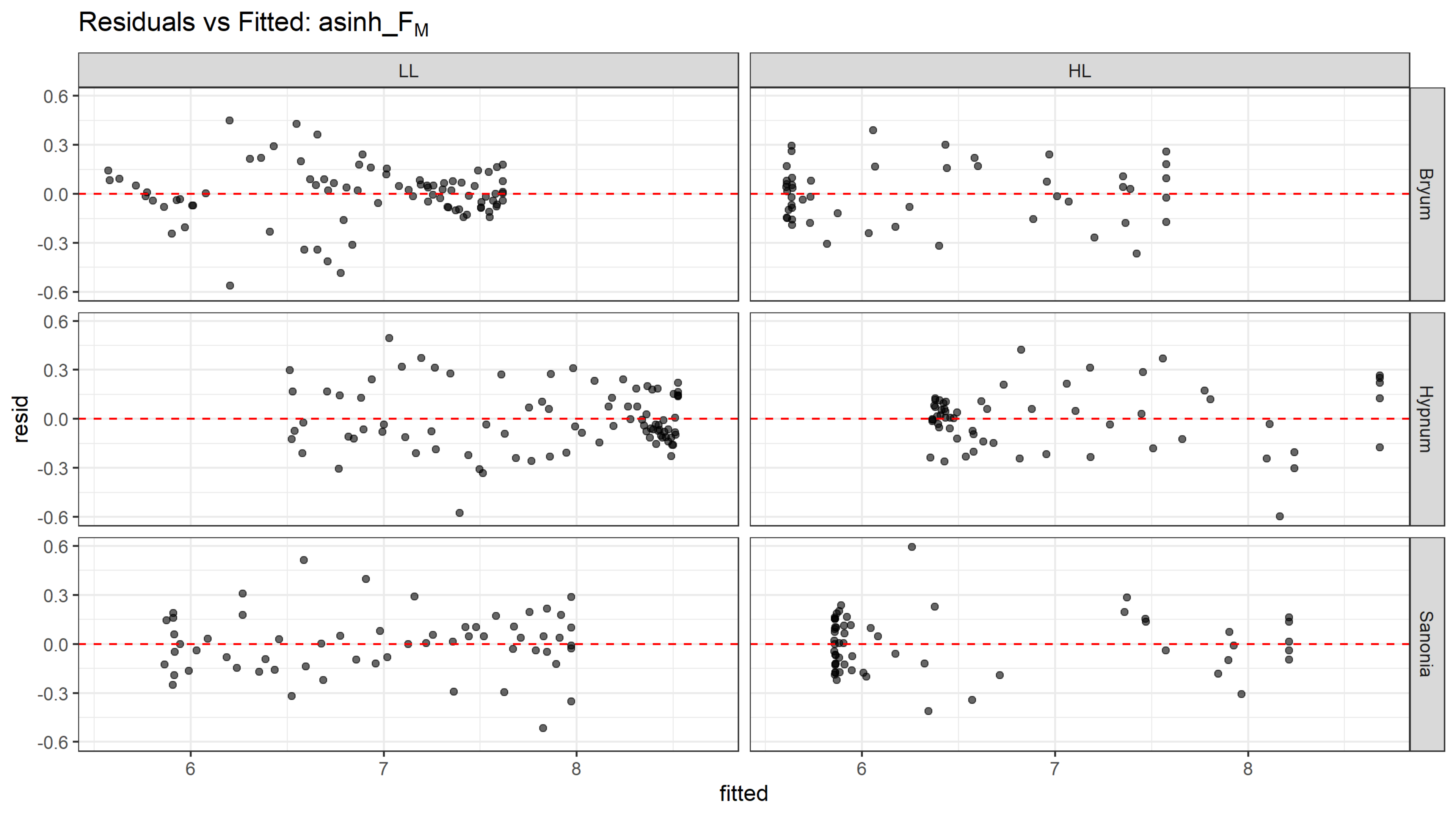

Residuals vs Fitted: asinh_F$_M$
LL
HL
Bryum
Hypnum
Sanonia
resid
fitted
0.6
0.3
0.0
-0.3
-0.6
6
7
8

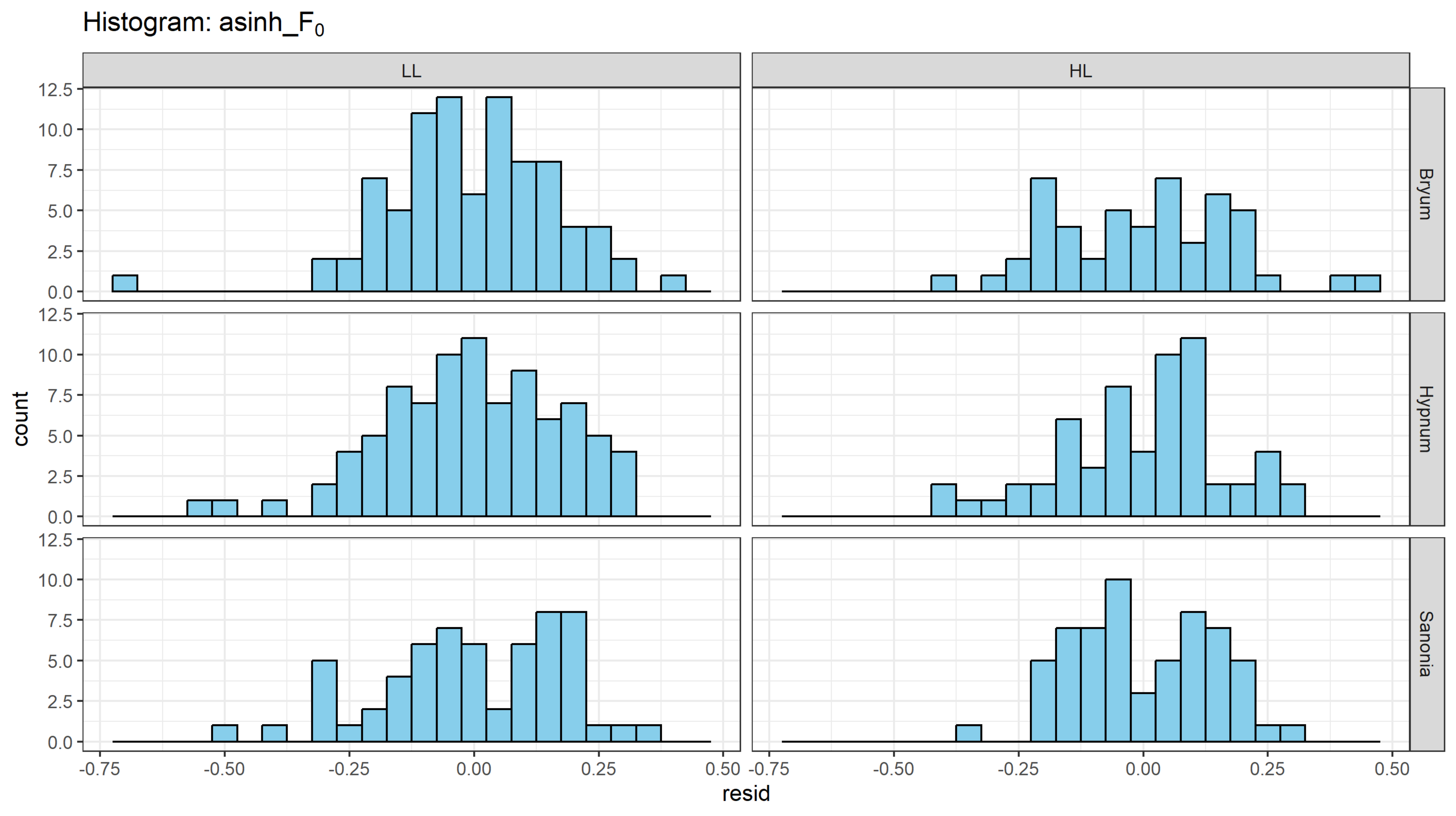

Histogram: asinh_F0
LL
HL
Bryum
Hypnum
Sanonia
count
resid
12.5
10.0
7.5
5.0
2.5
0.0
-0.75
-0.50
-0.25
0.00
0.25
0.50

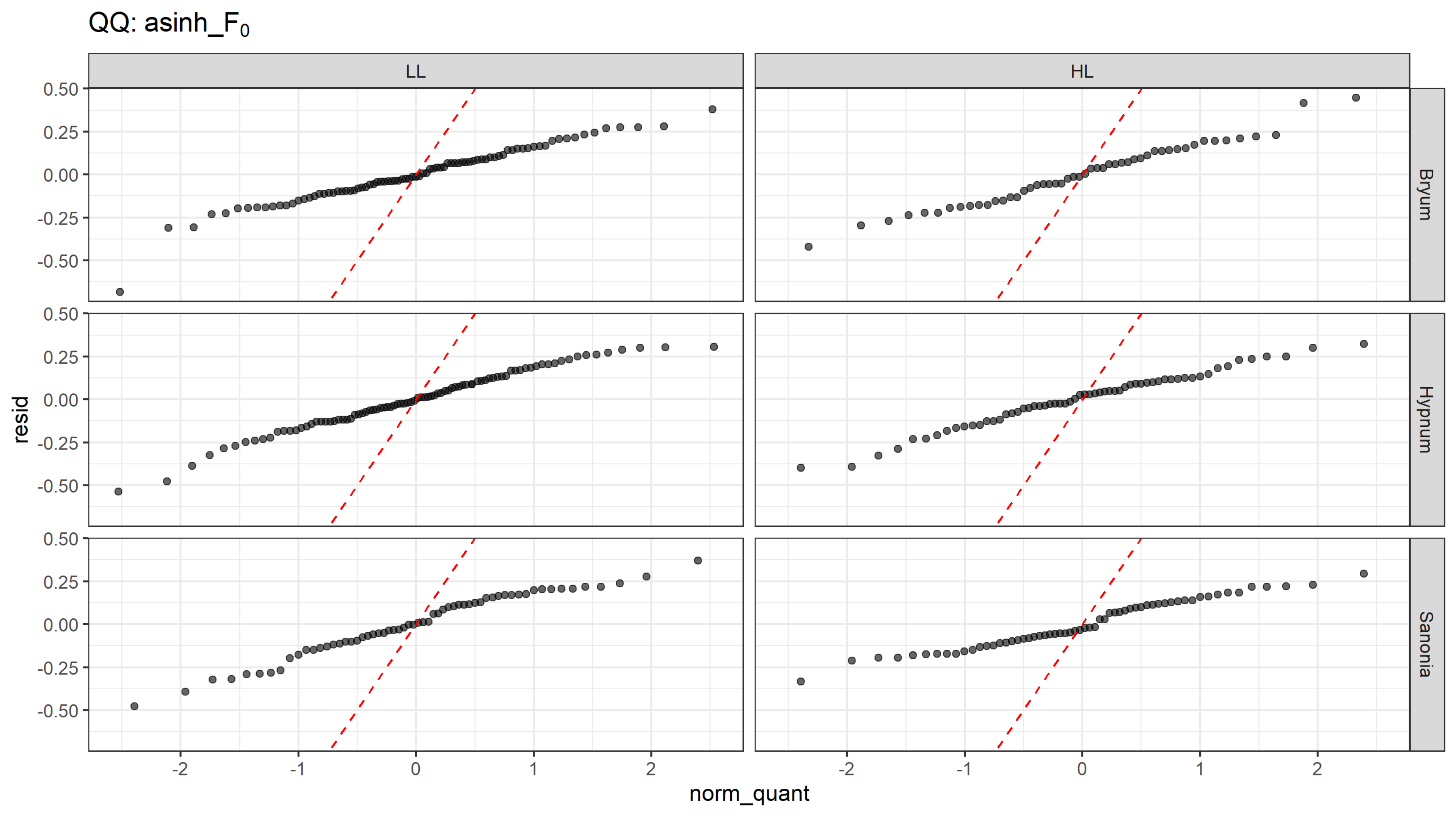

QQ: asinh_F0
LL
HL
Bryum
Hypnum
Sanonia
resid
norm_quant
0.50
0.25
0.00
-0.25
-0.50
-2
-1
0
1
2

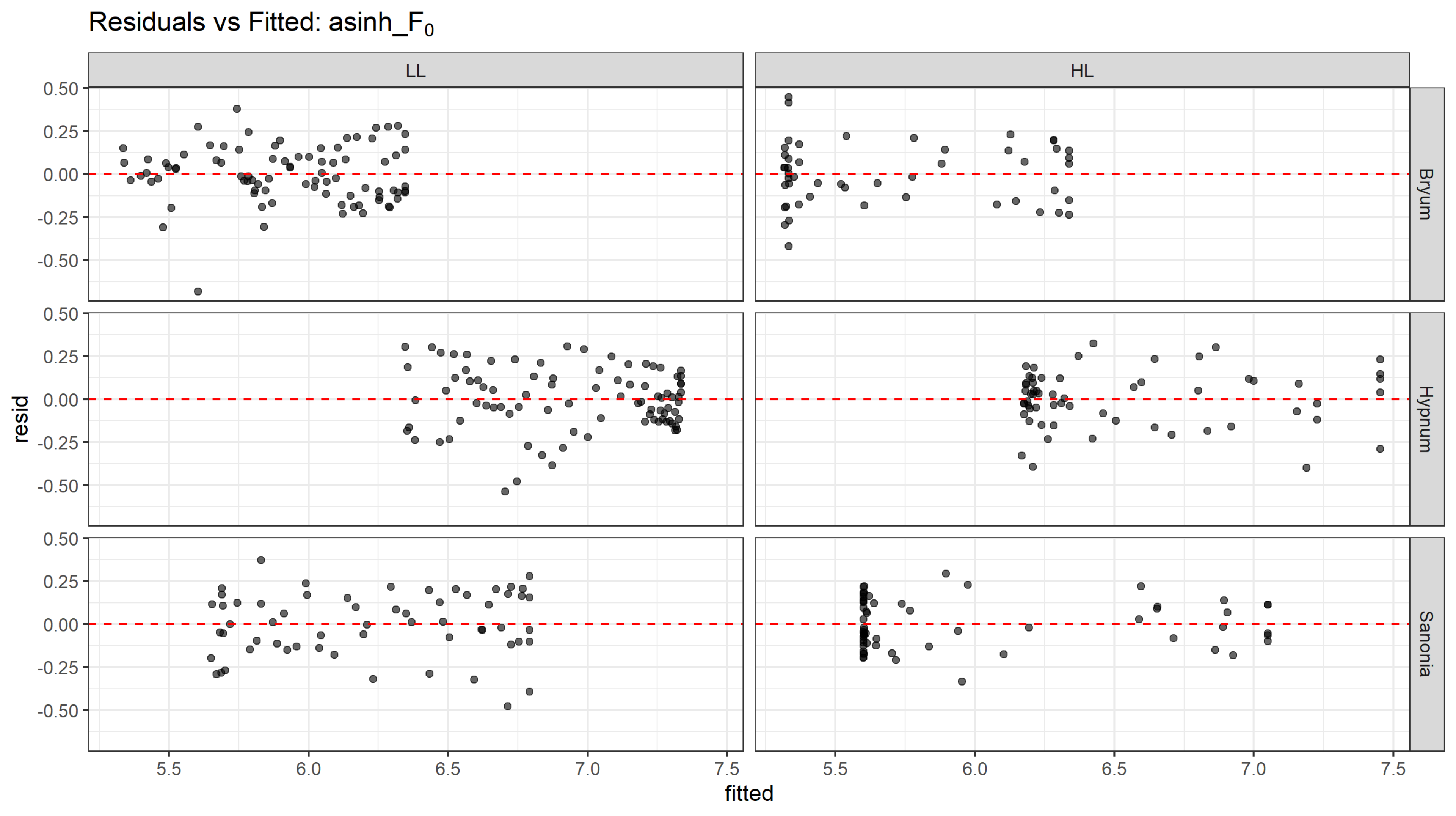

Residuals vs Fitted: asinh_F_0
LL
HL
Bryum
Hypnum
Sanonia
resid
fitted
0.50
0.25
0.00
-0.25
-0.50
5.5
6.0
6.5
7.0
7.5

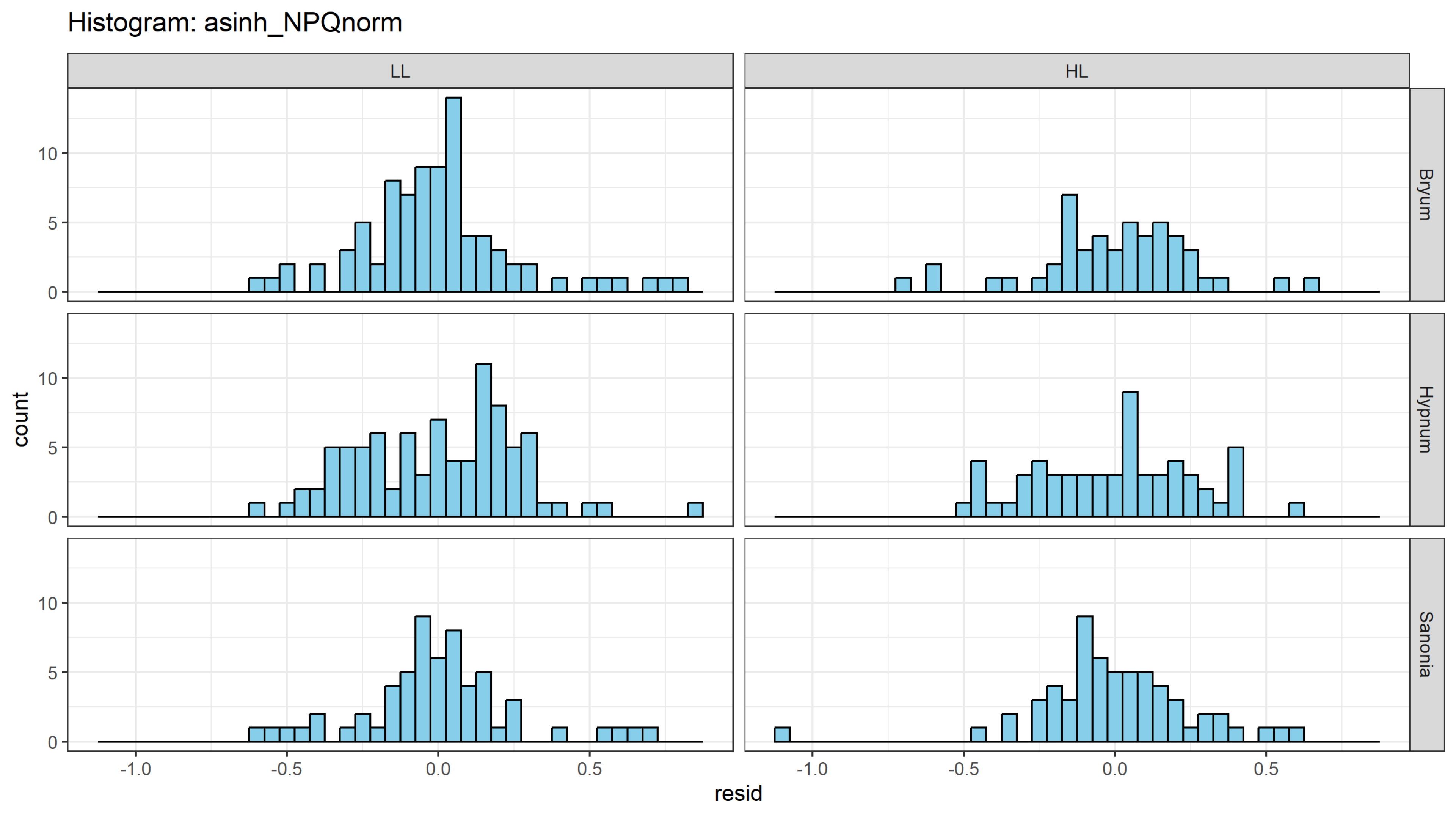

Histogram: asinh_NPQnorm
LL
HL
Bryum
Hypnum
Sanonia
count
resid
0
5
10
-1.0
-0.5
0.0
0.5

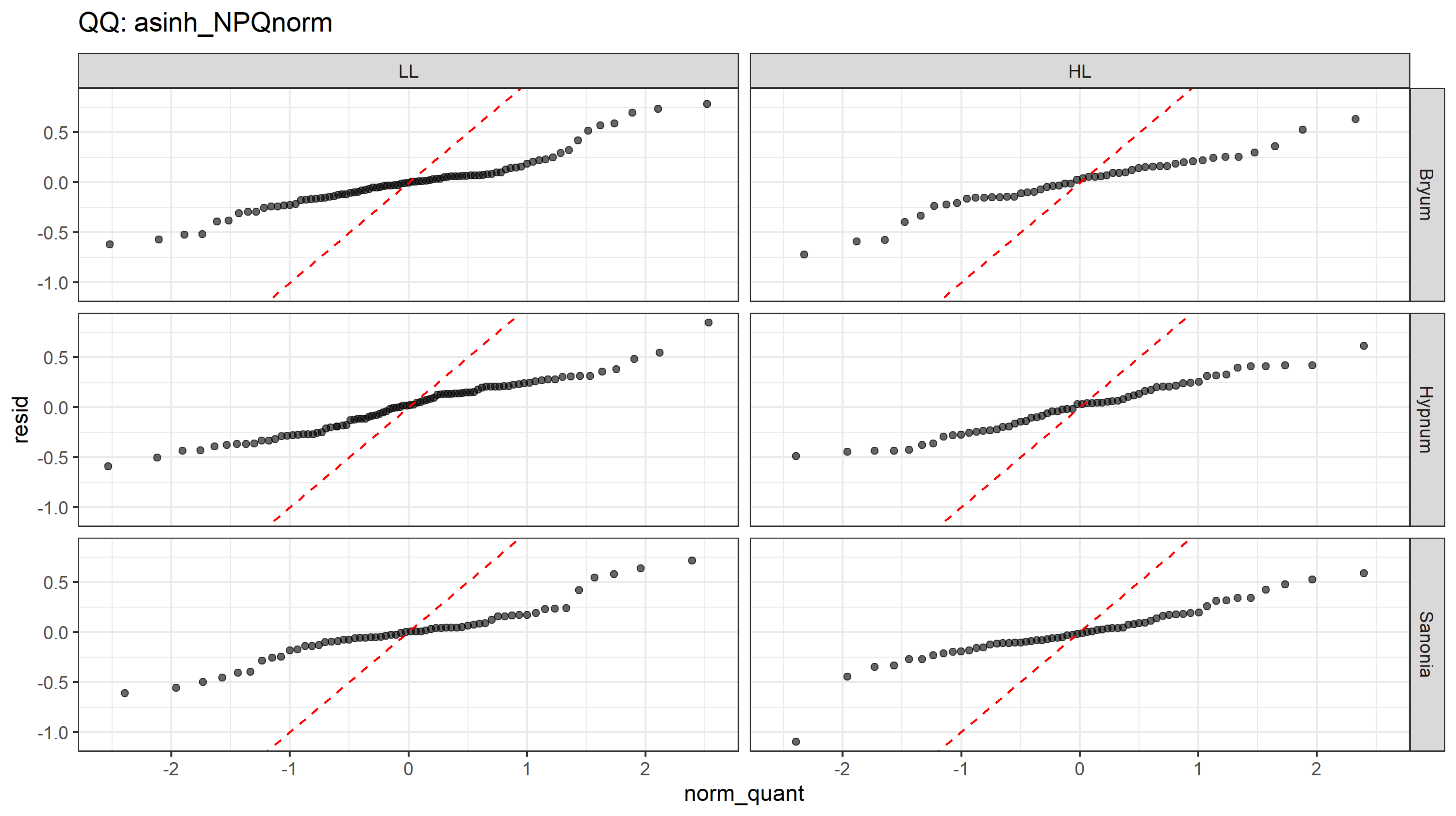
QQ: asinh_NPQnorm
LL
HL
Bryum
Hypnum
Sanonia
resid
norm_quant
0.5
0.0
-0.5
-1.0
-2
-1
0
1
2

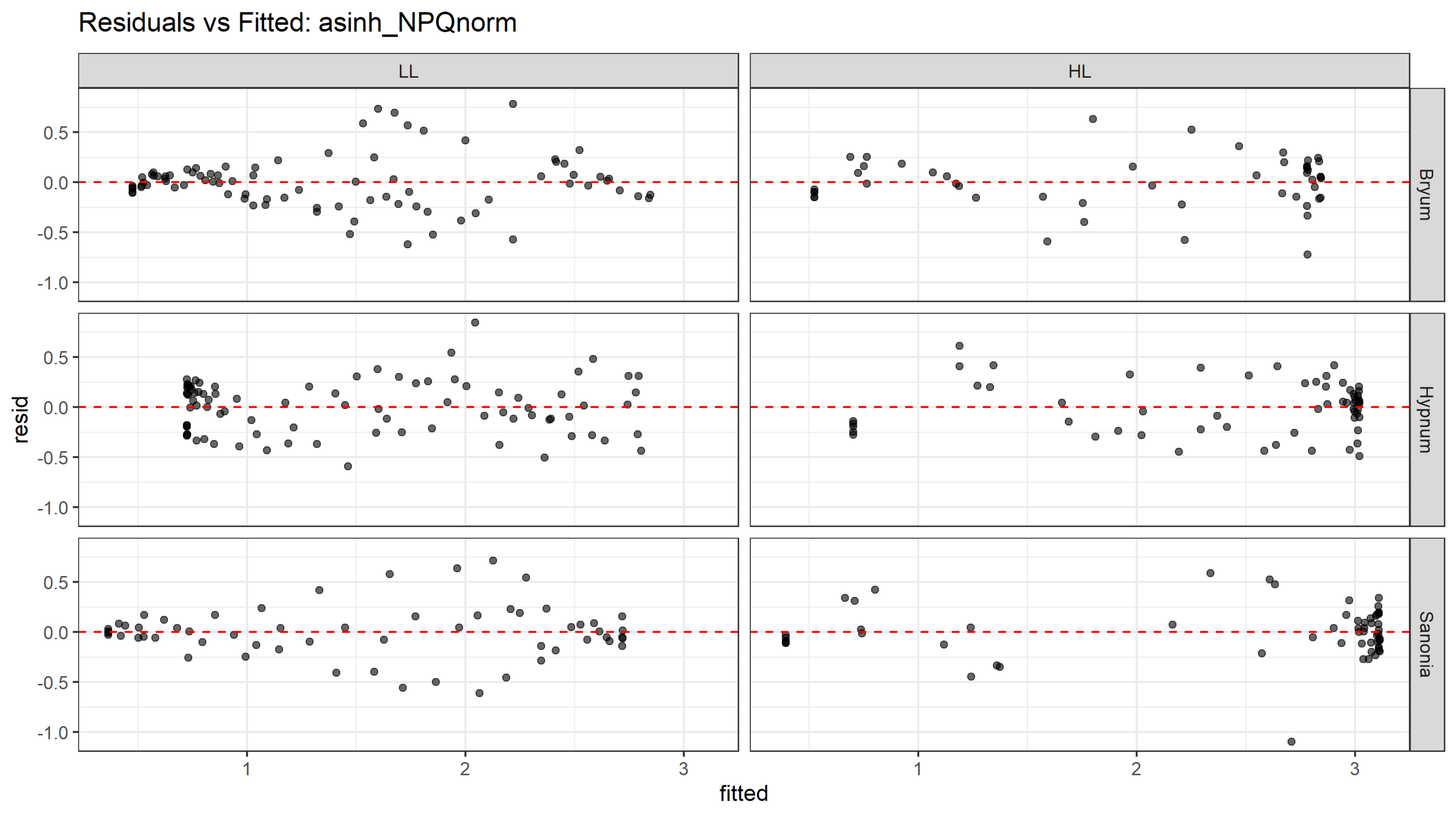

Residuals vs Fitted: asinh_NPQnorm
LL
HL
Bryum
Hypnum
Sanonia
resid
fitted
0.5
0.0
-0.5
-1.0
1
2
3

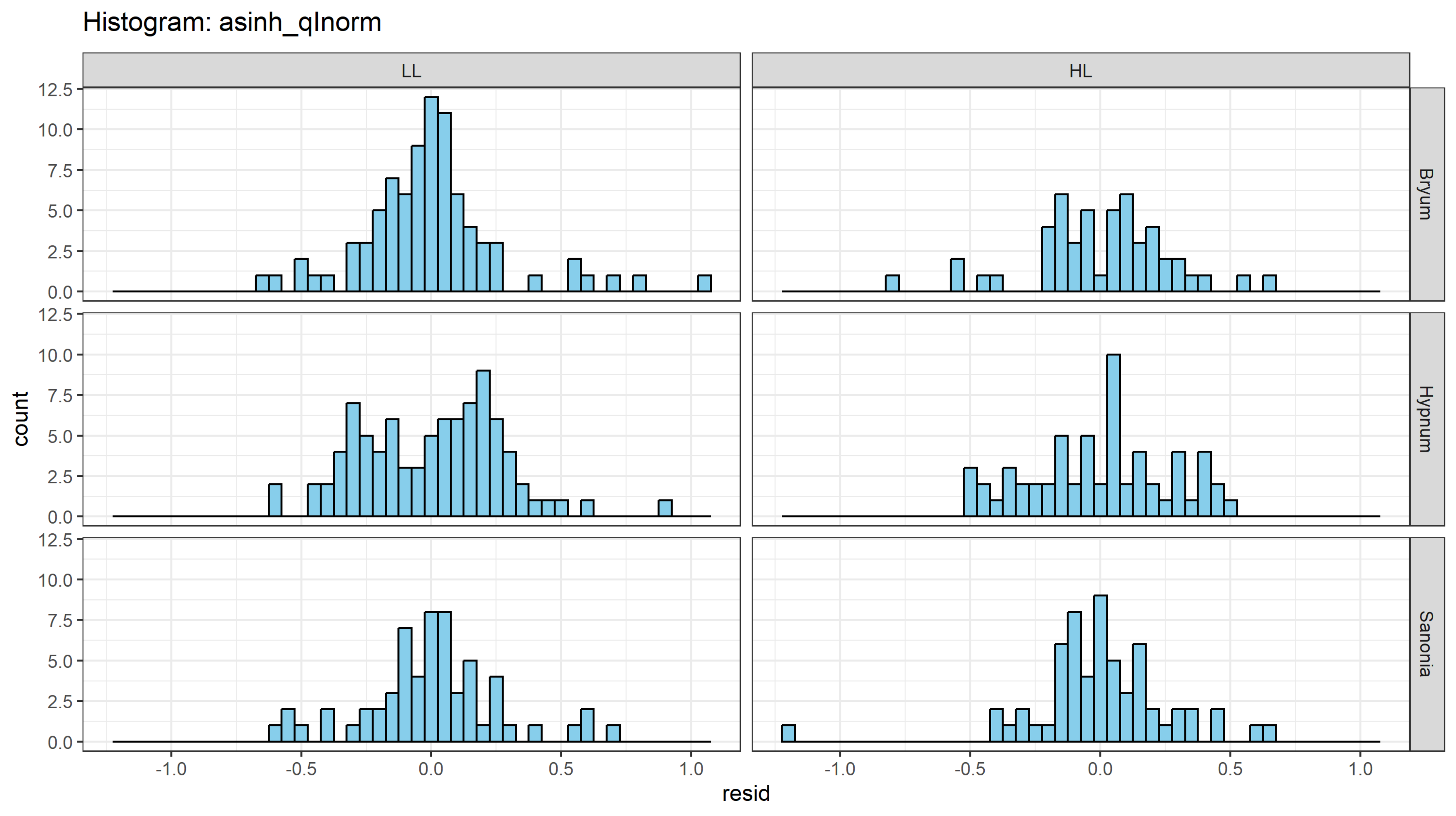
Histogram: asinh_qlnorm
LL
HL
Bryum
Hypnum
Sanonia
count
resid
12.5
10.0
7.5
5.0
2.5
0.0
-1.0
-0.5
0.0
0.5
1.0

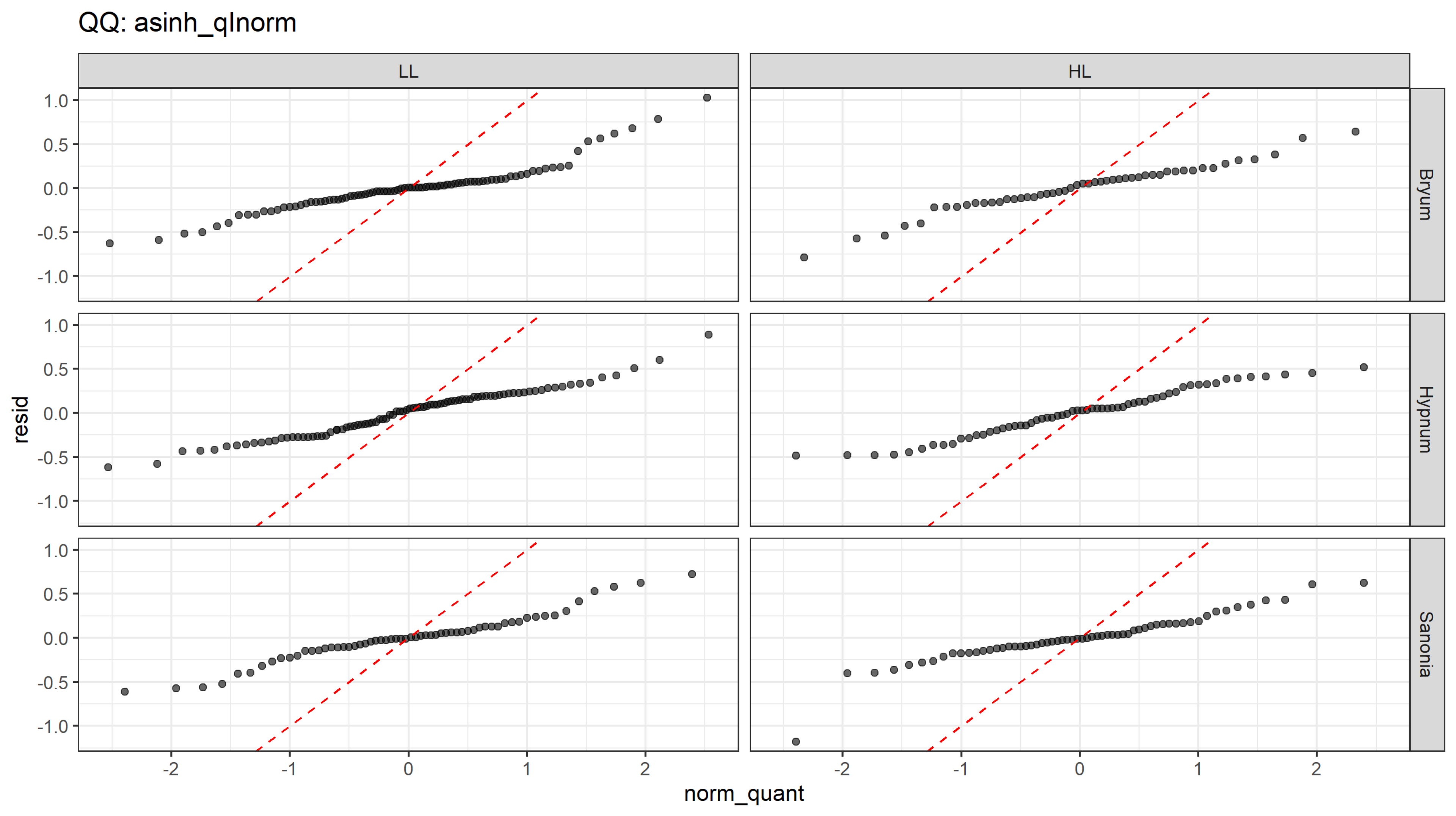
QQ: asinh_qlnorm
LL
HL
Bryum
Hypnum
Sanonia
resid
norm_quant
1.0
0.5
0.0
-0.5
-1.0
-2
-1
0
1
2

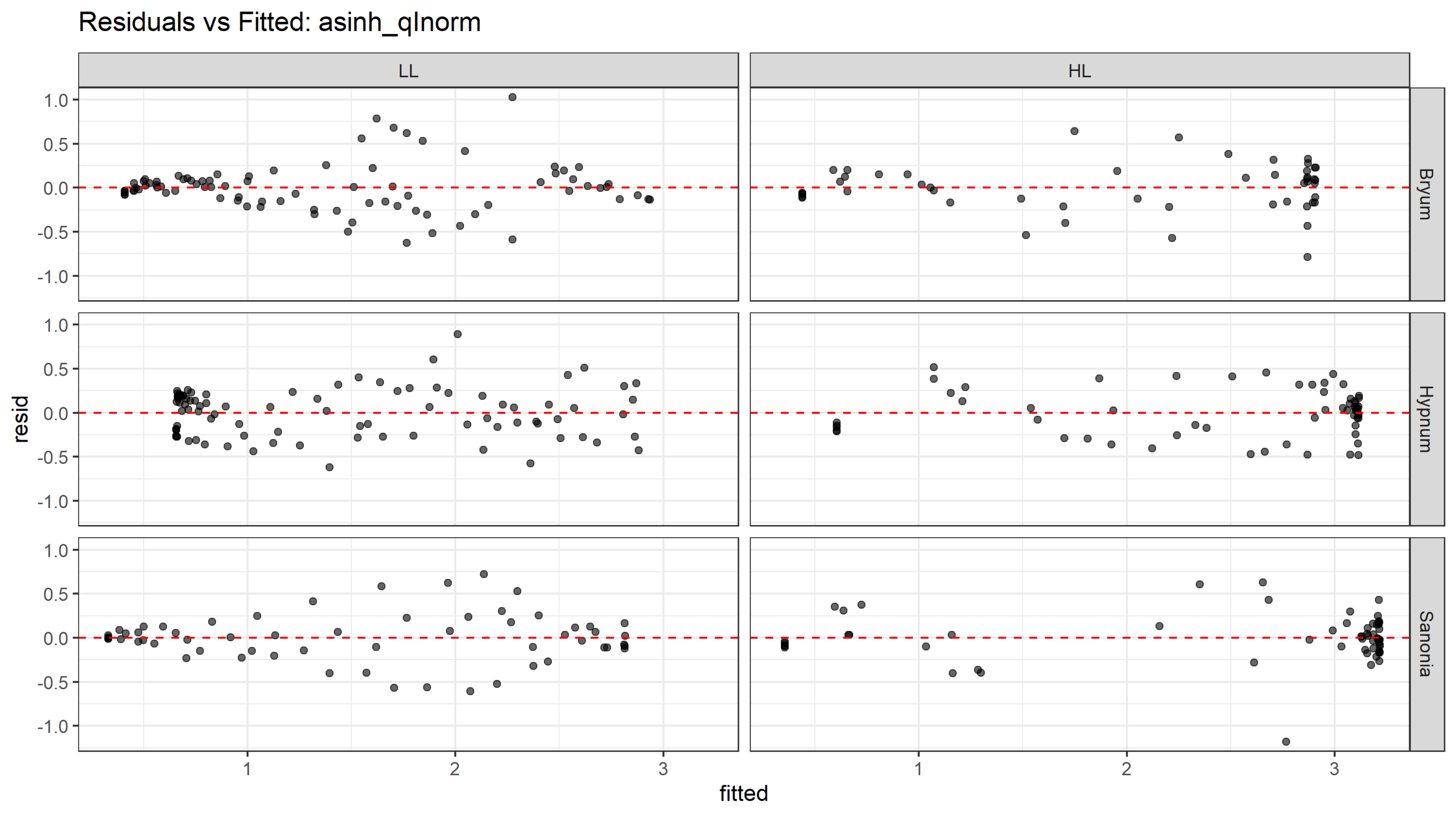
Residuals vs Fitted: asinh_qlnorm
LL
HL
Bryum
Hypnum
Sanonia
resid
fitted
1.0
0.5
0.0
-0.5
-1.0
1
2
3

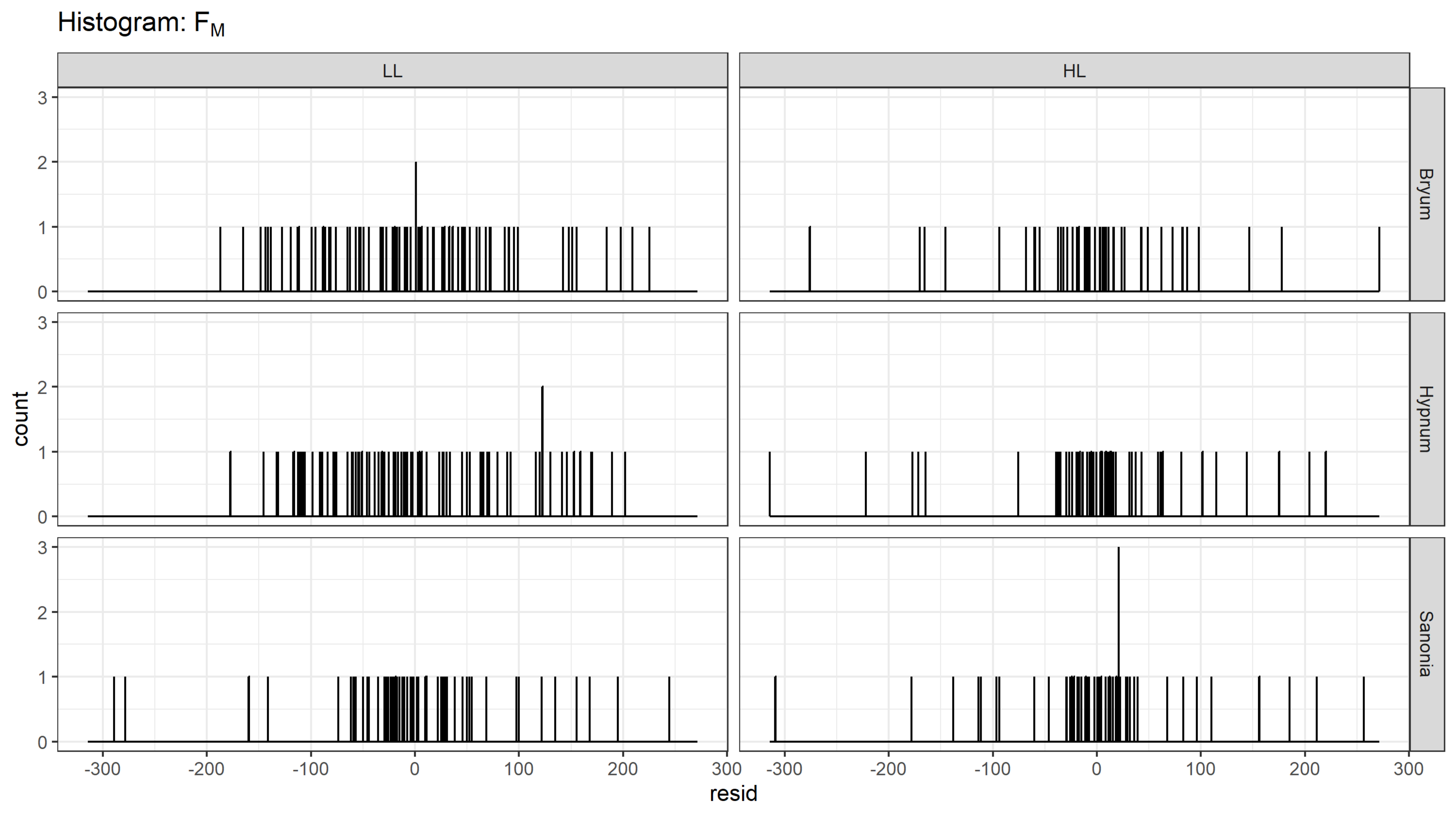
Histogram: F_M
LL
HL
Bryum
Hypnum
Sanonia
count
resid
0
1
2
3
-300
-200
-100
0
100
200
300

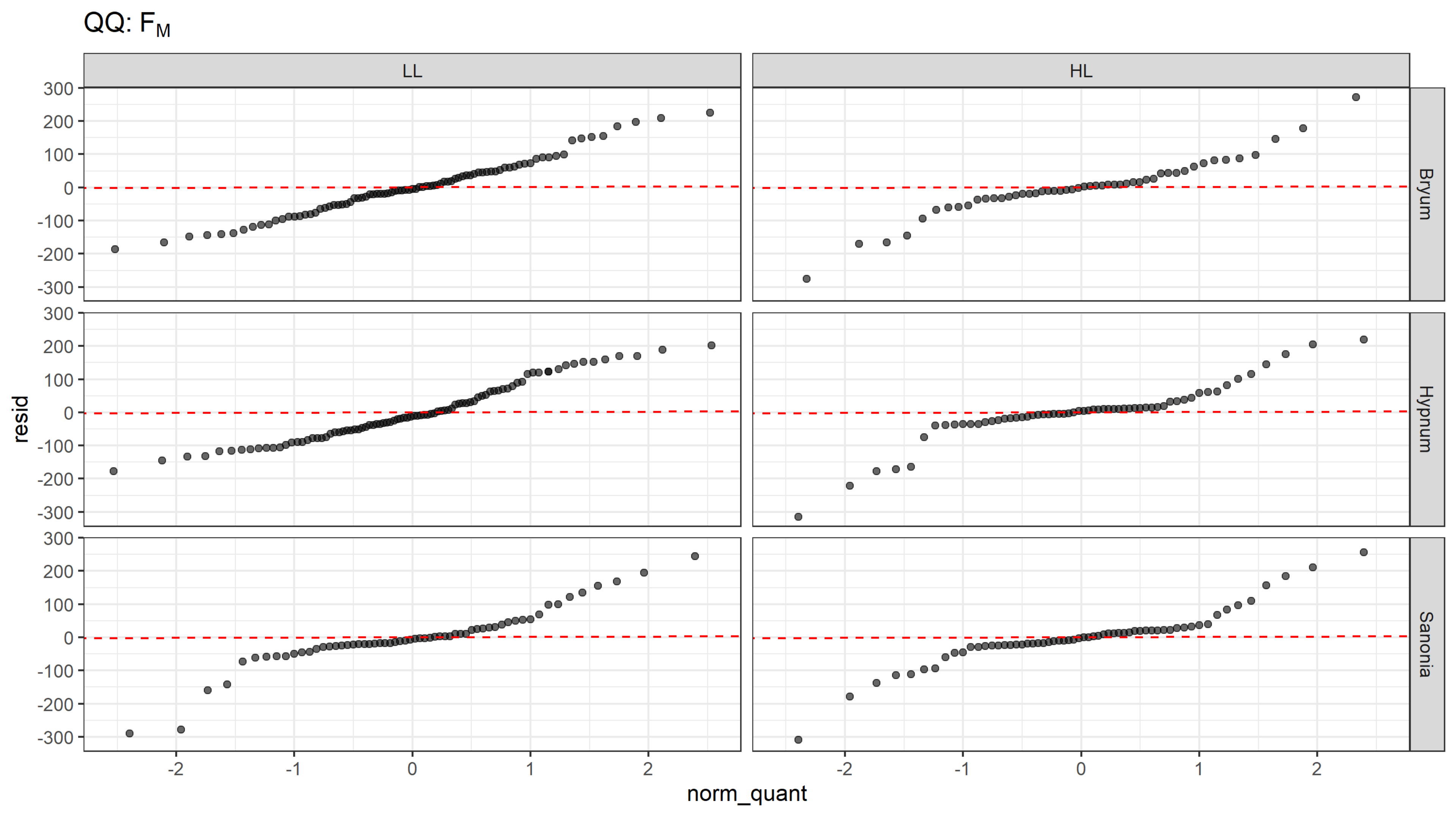
QQ: F_M
LL
HL
Bryum
Hypnum
Sanonia
resid
norm_quant
300
200
100
0
-100
-200
-300
-2
-1
0
1
2

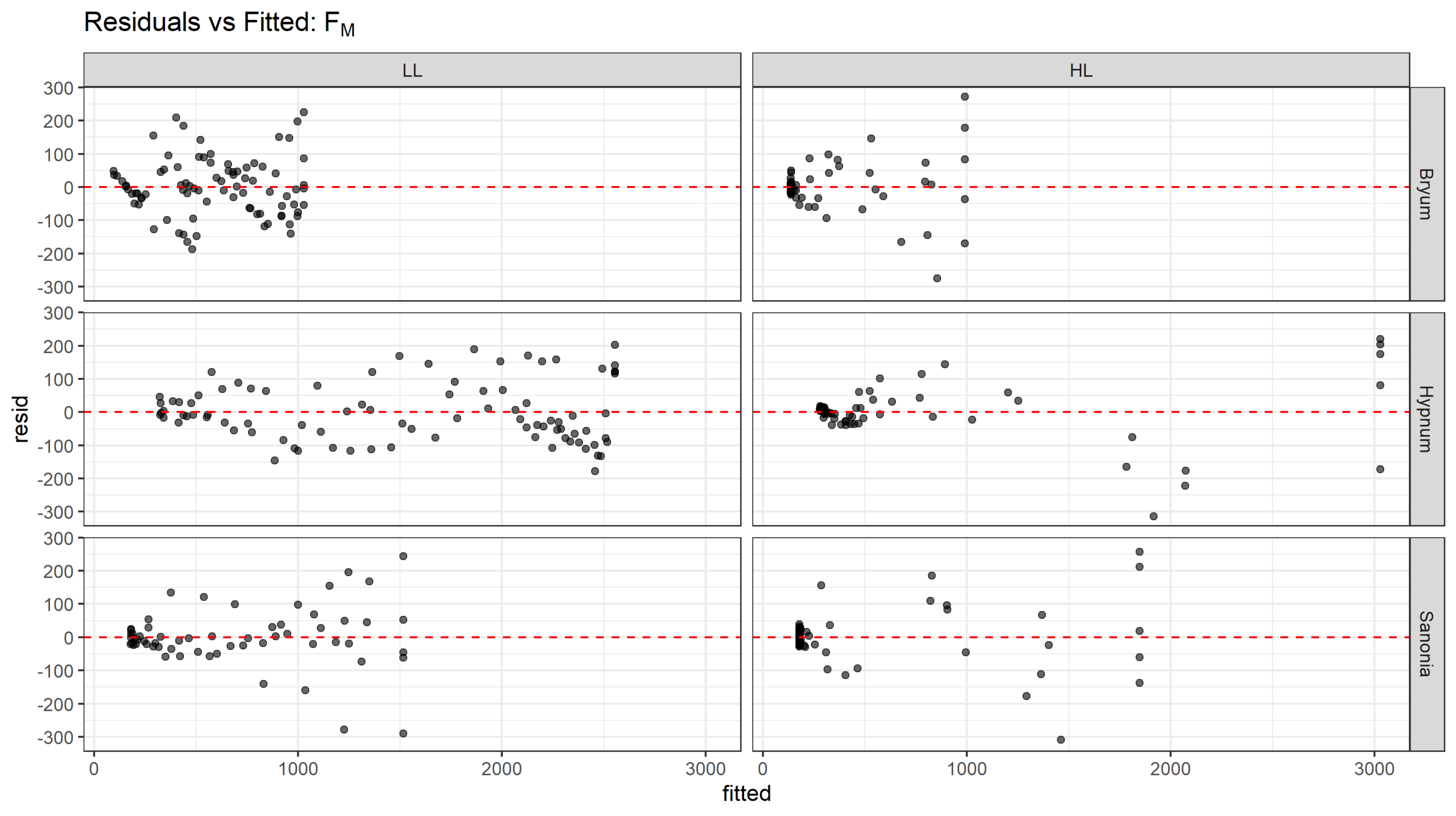

Residuals vs Fitted: F_M
LL
HL
Bryum
Hypnum
Sanonia
resid
fitted
300
200
100
0
-100
-200
-300
0
1000
2000
3000

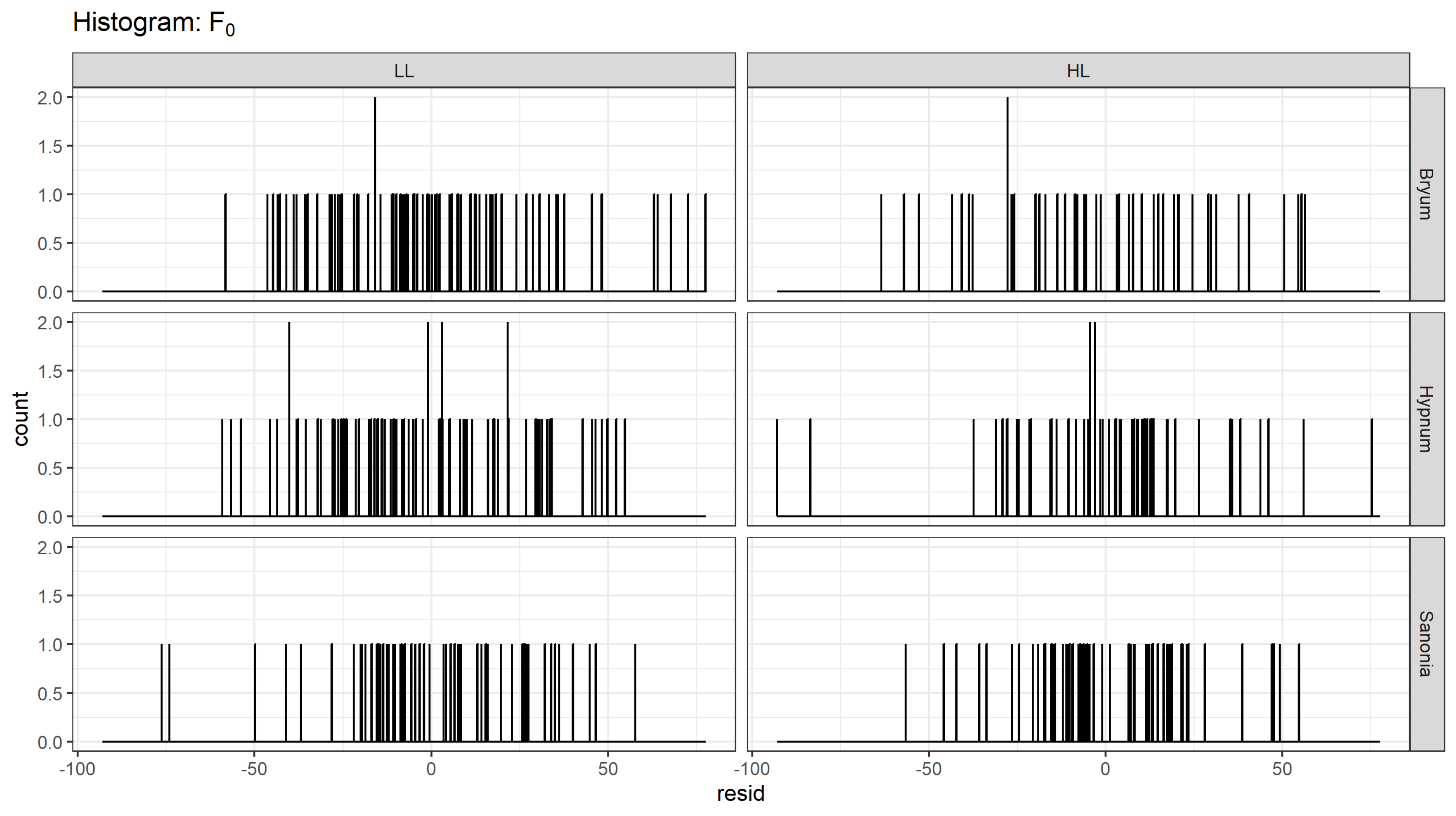

Histogram: F_0
LL
HL
Bryum
Hypnum
Sanonia
count
resid
2.0
1.5
1.0
0.5
0.0
-100
-50
0
50

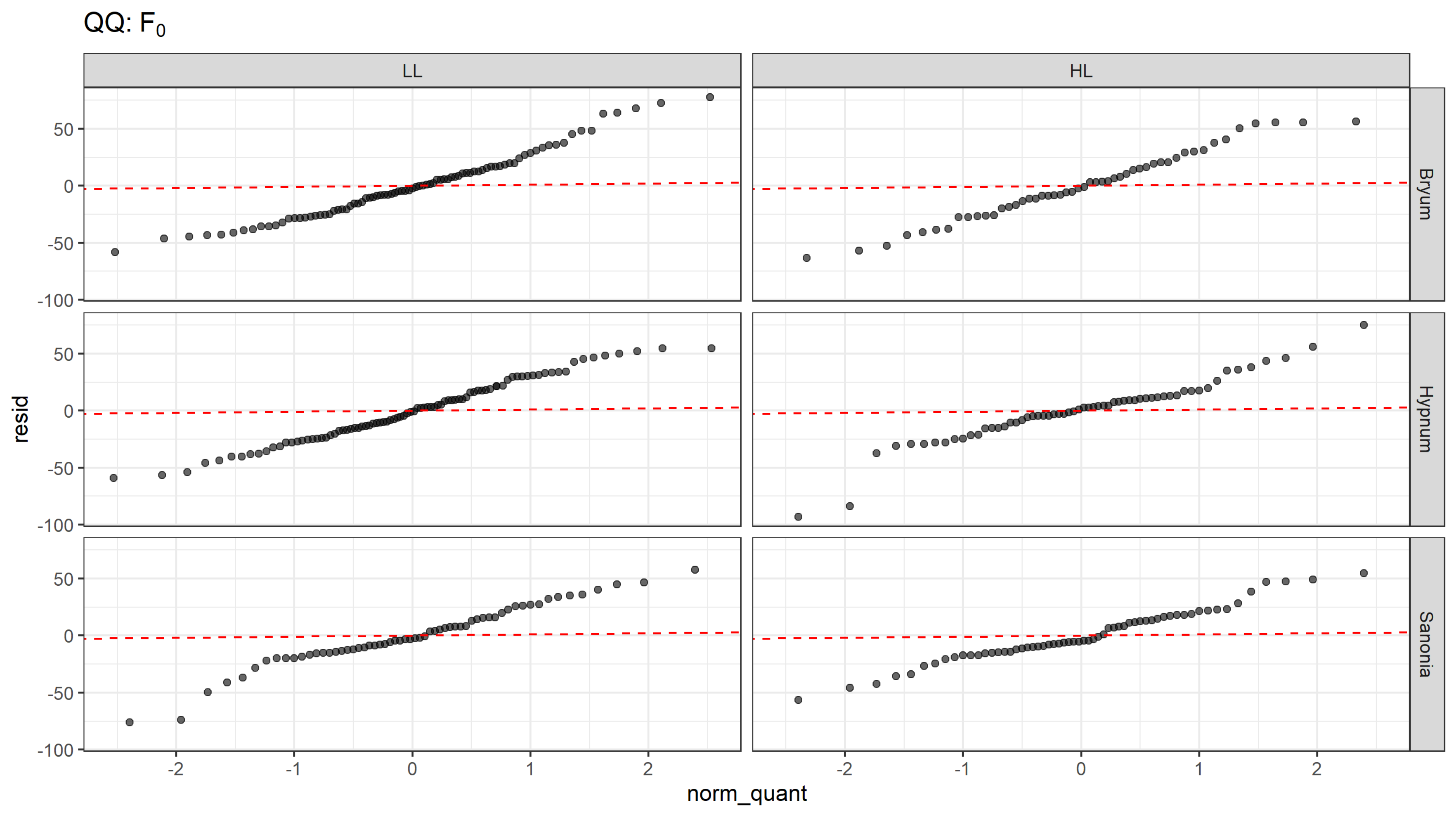
QQ: F_0
LL
HL
Bryum
Hypnum
Sanonia
resid
norm_quant
50
0
-50
-100
-2
-1
0
1
2

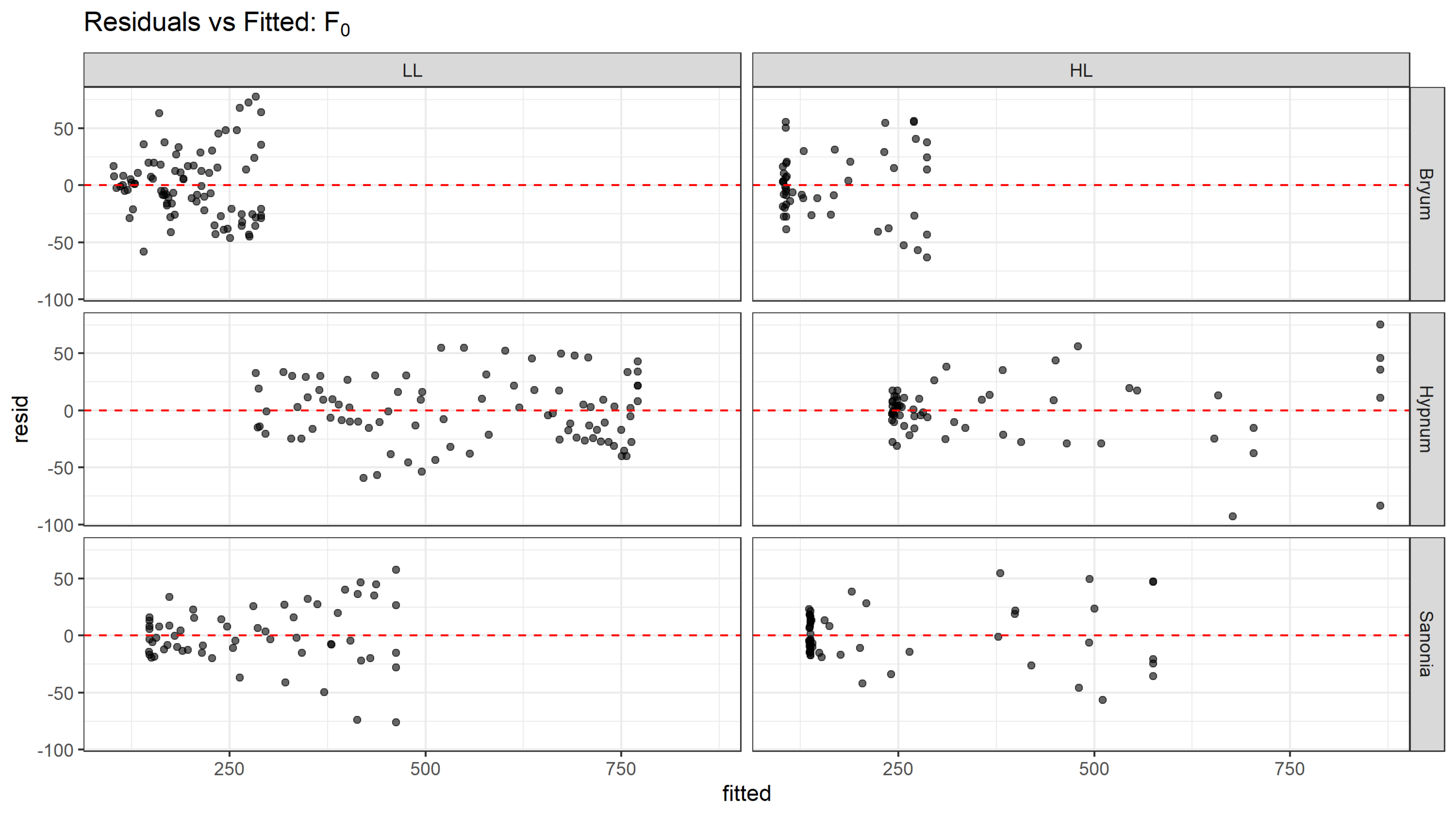

Residuals vs Fitted: F_0
LL
HL
Bryum
Hypnum
Sanonia
resid
fitted
50
0
-50
-100
250
500
750

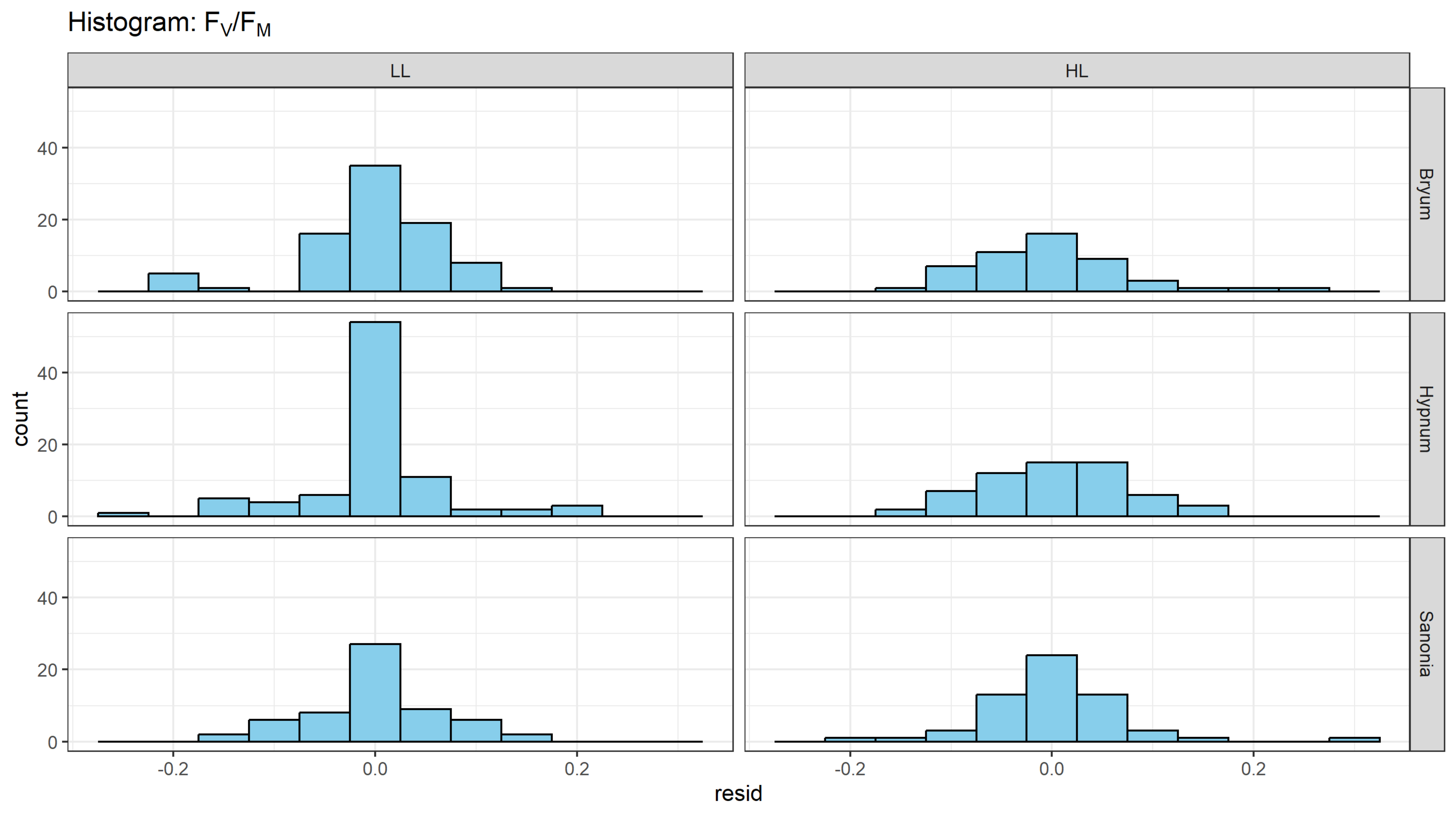

Histogram: F_V/F_M
LL
HL
Bryum
Hypnum
Sanonia
count
resid
0
20
40
-0.2
0.0
0.2

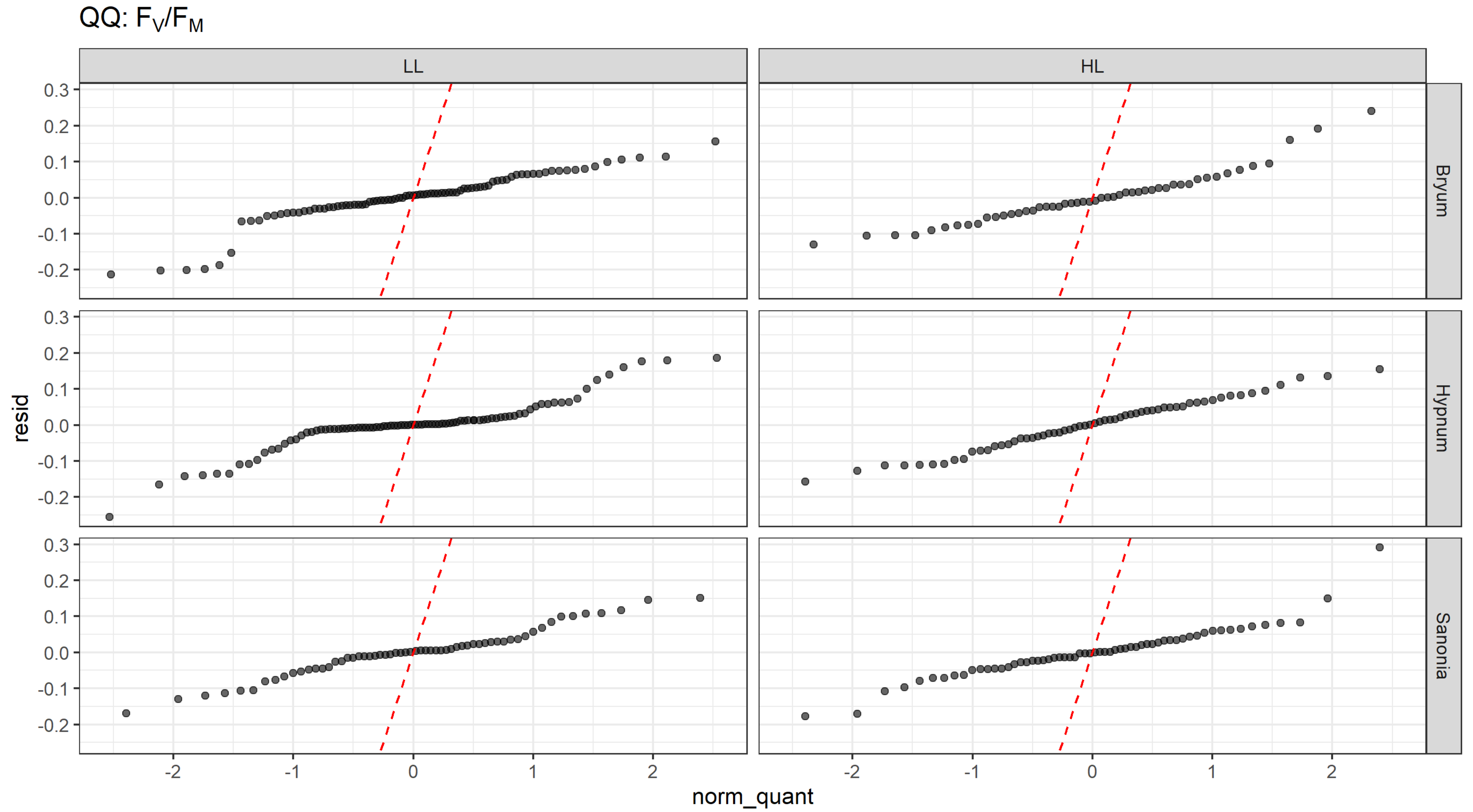
QQ: FV/FM
LL
HL
Bryum
Hypnum
Sanonia
resid
norm_quant
0.3
0.2
0.1
0.0
-0.1
-0.2
-2
-1
0
1
2

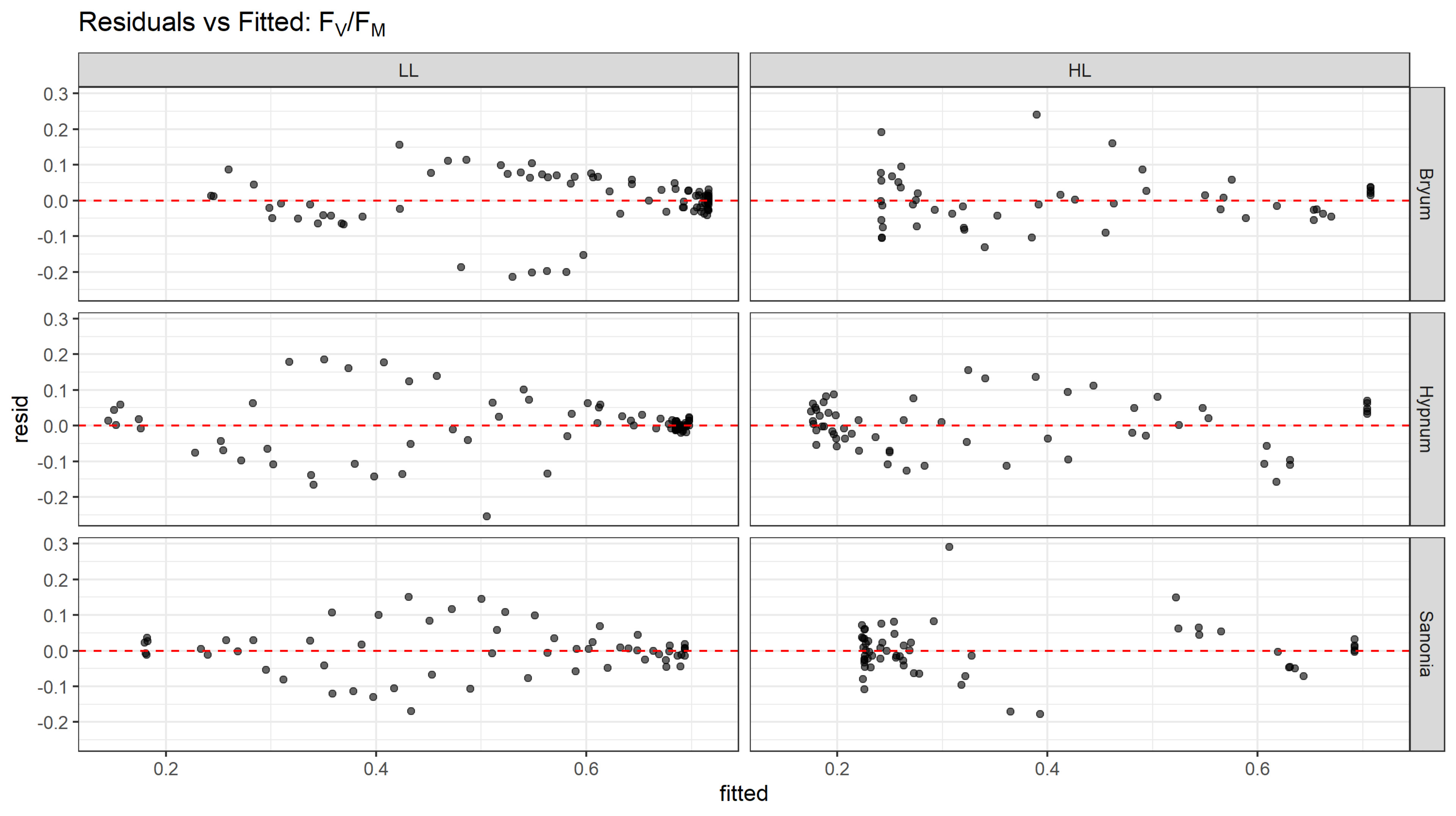

Residuals vs Fitted: F_V/F_M
LL
HL
Bryum
Hypnum
Sanonia
resid
fitted
0.3
0.2
0.1
0.0
-0.1
-0.2
0.2
0.4
0.6

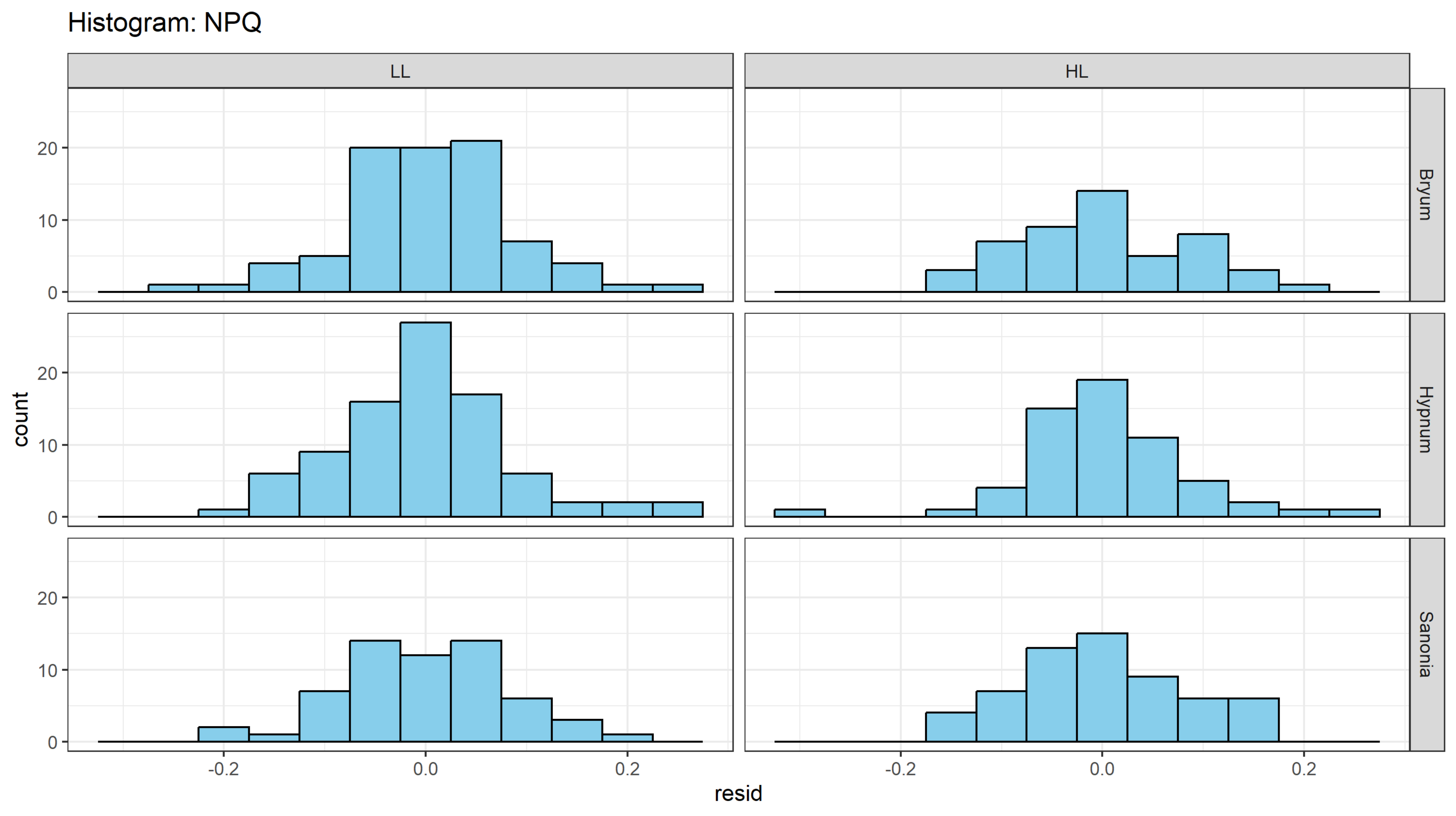
Histogram: NPQ
LL
HL
Bryum
Hypnum
Sanonia
count
resid
0
10
20
-0.2
0.0
0.2

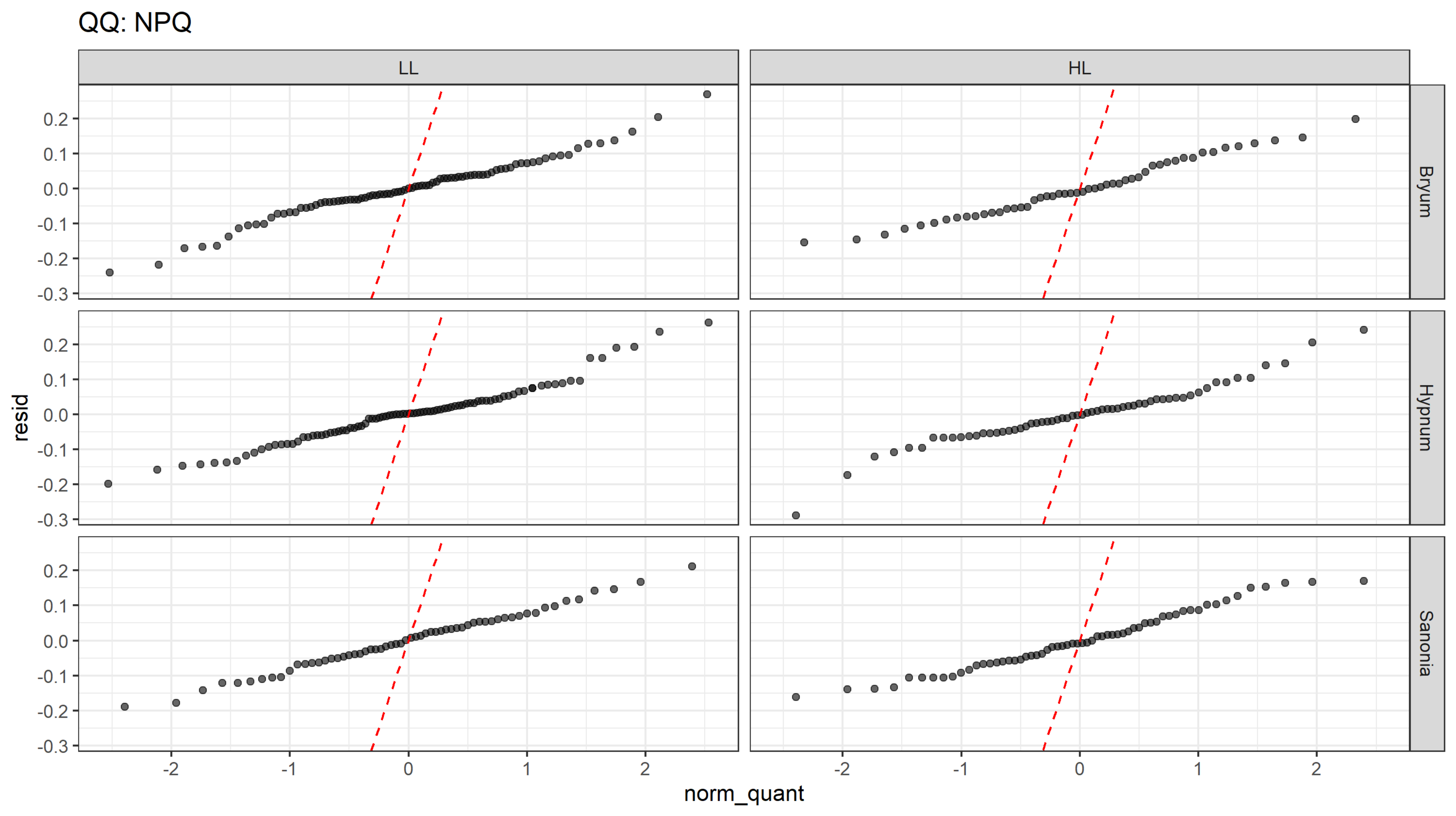

QQ: NPQ
LL
HL
Bryum
Hypnum
Sanonia
resid
norm_quant
0.2
0.1
0.0
-0.1
-0.2
-0.3
-2
-1
0
1
2

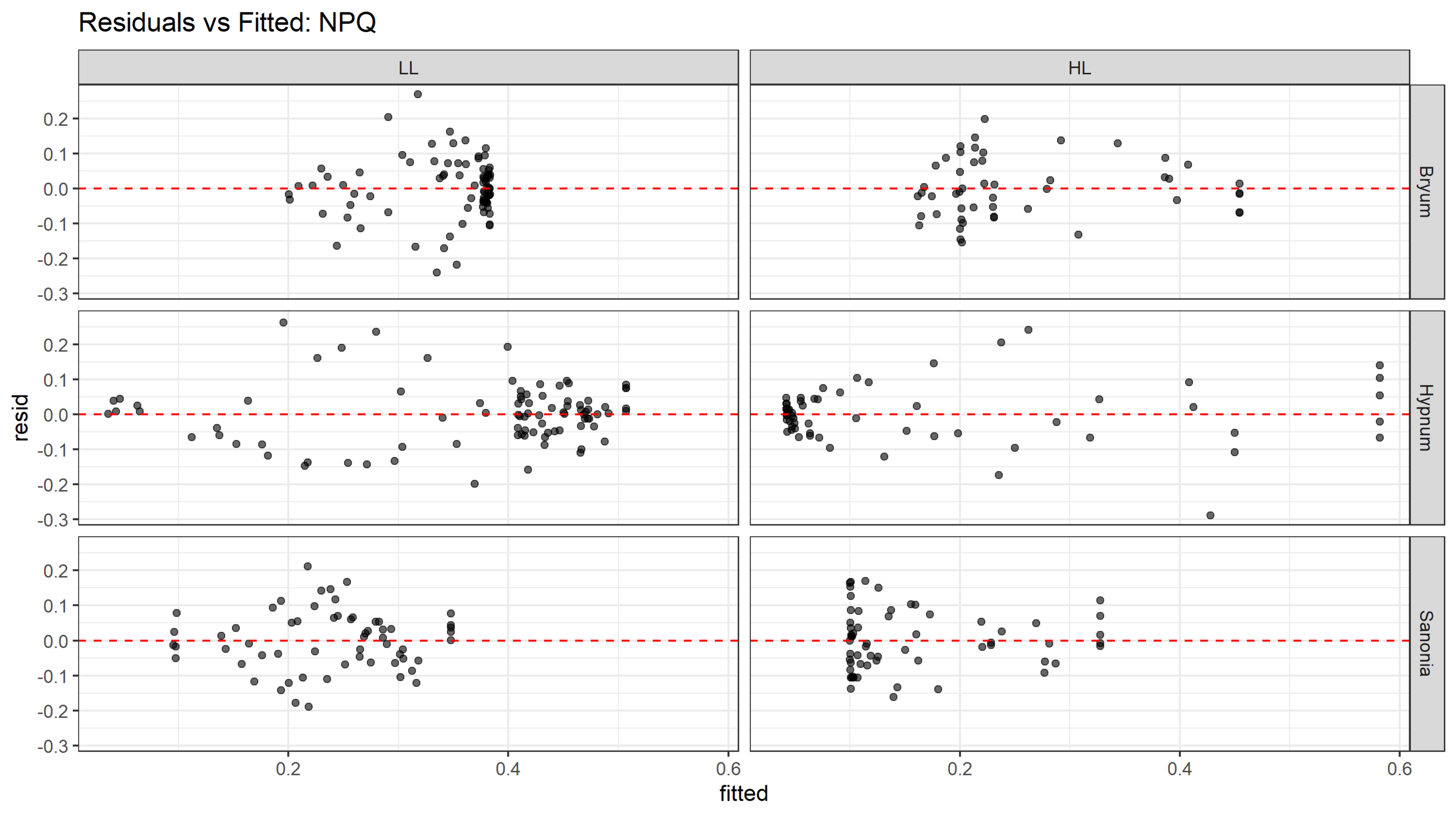

Residuals vs Fitted: NPQ
LL
HL
Bryum
Hypnum
Sanonia
resid
fitted
0.2
0.1
0.0
-0.1
-0.2
-0.3
0.2
0.4
0.6

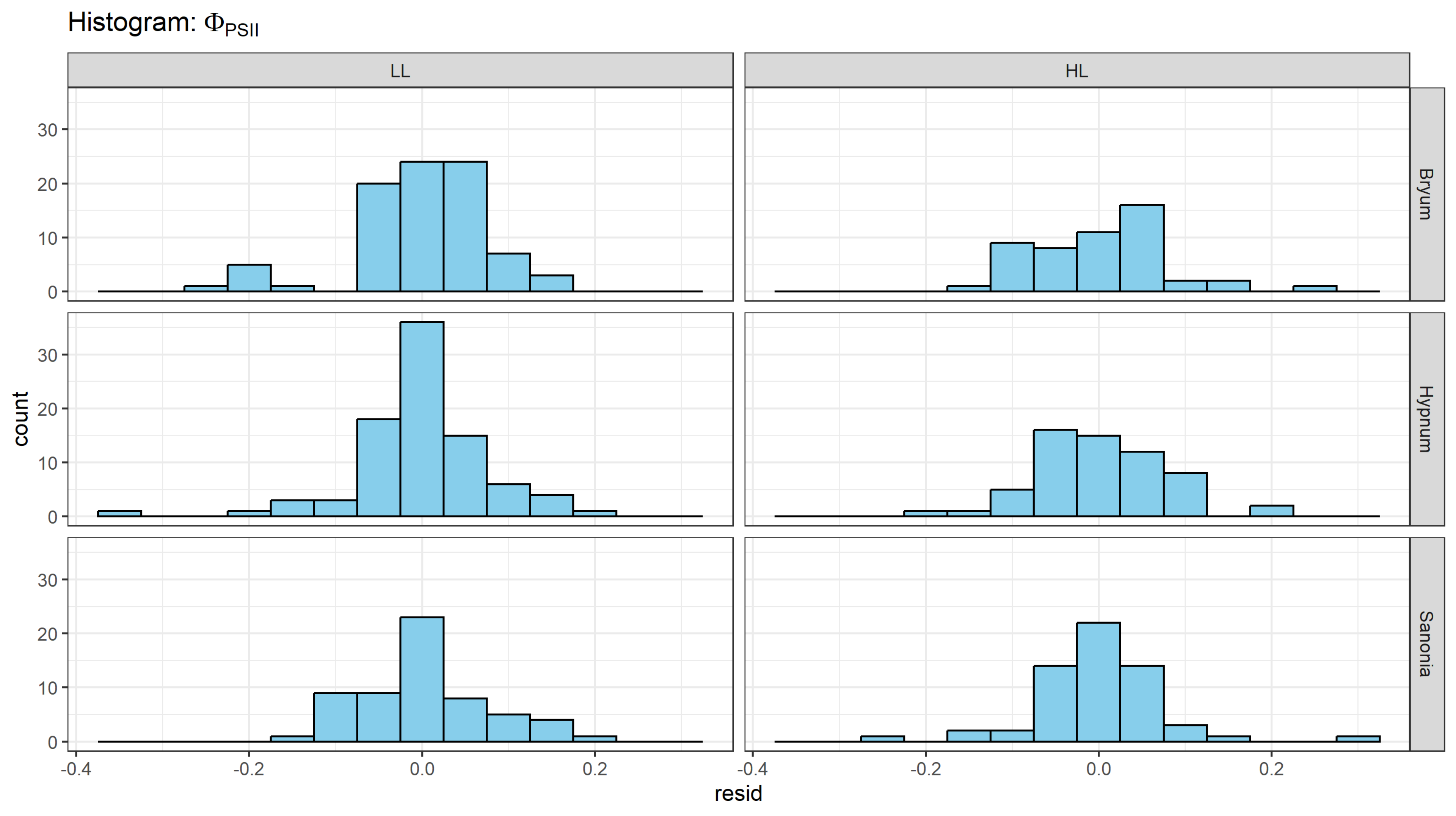

Histogram: Φ_{PSII}
LL
HL
Bryum
Hypnum
Sanonia
count
resid
0
10
20
30
-0.4
-0.2
0.0
0.2

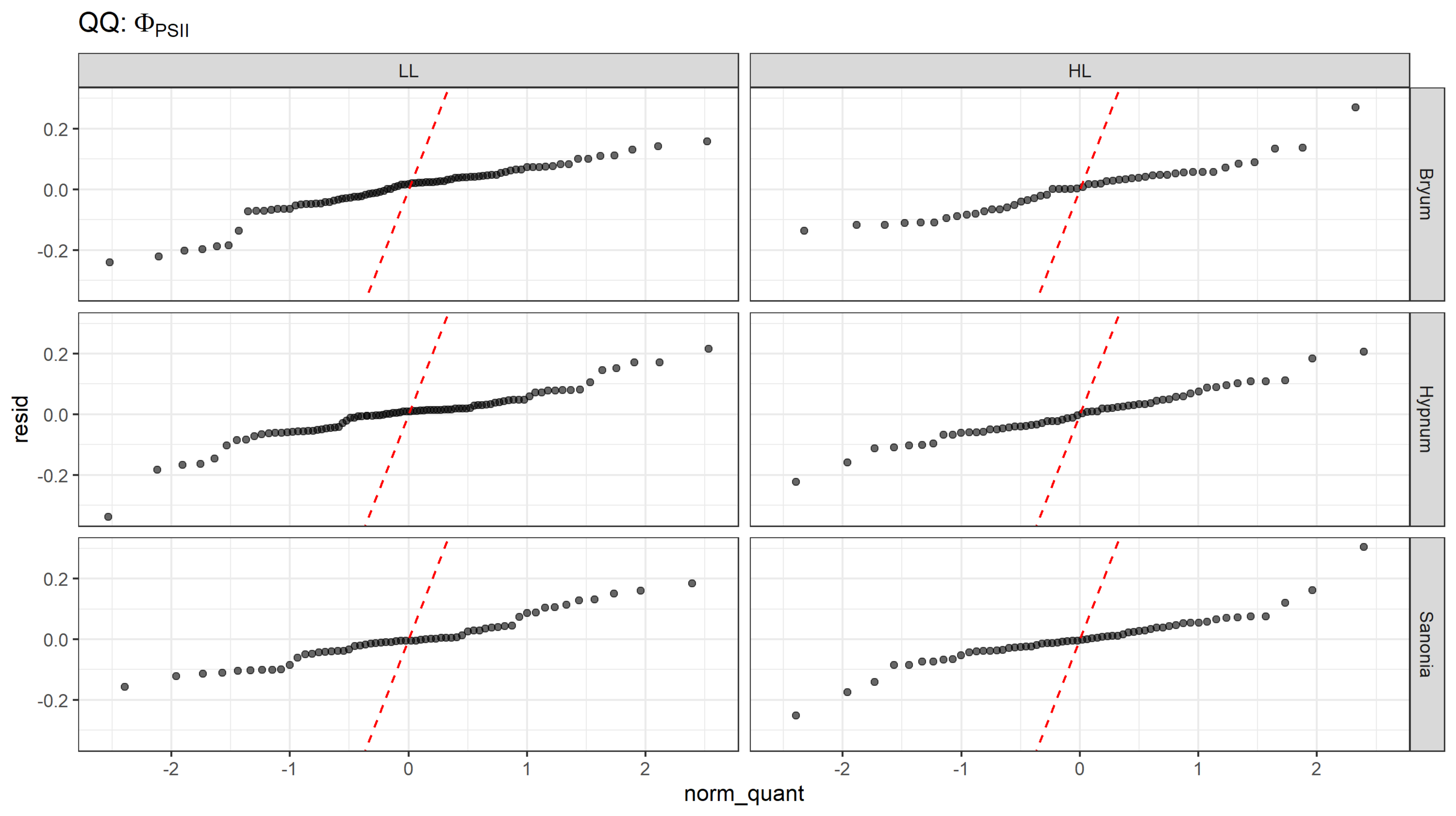
QQ: Φ_{PSII}
LL
HL
Bryum
Hypnum
Sanonia
resid
norm_quant
0.2
0.0
-0.2
-2
-1
0
1
2

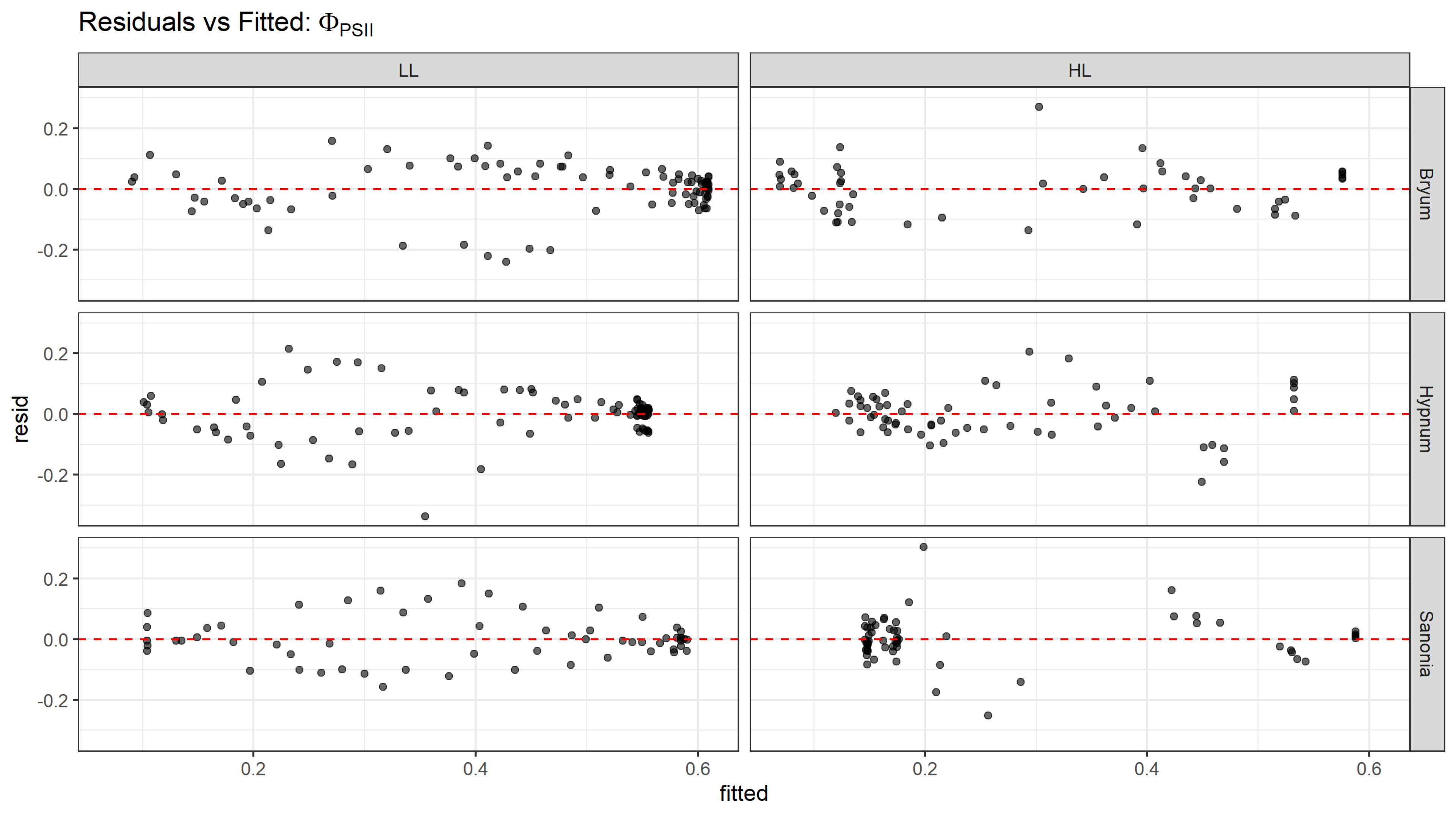

Residuals vs Fitted: Φ_{PSII}
LL
HL
Bryum
Hypnum
Sanonia
resid
fitted
0.2
0.0
-0.2
0.4
0.6

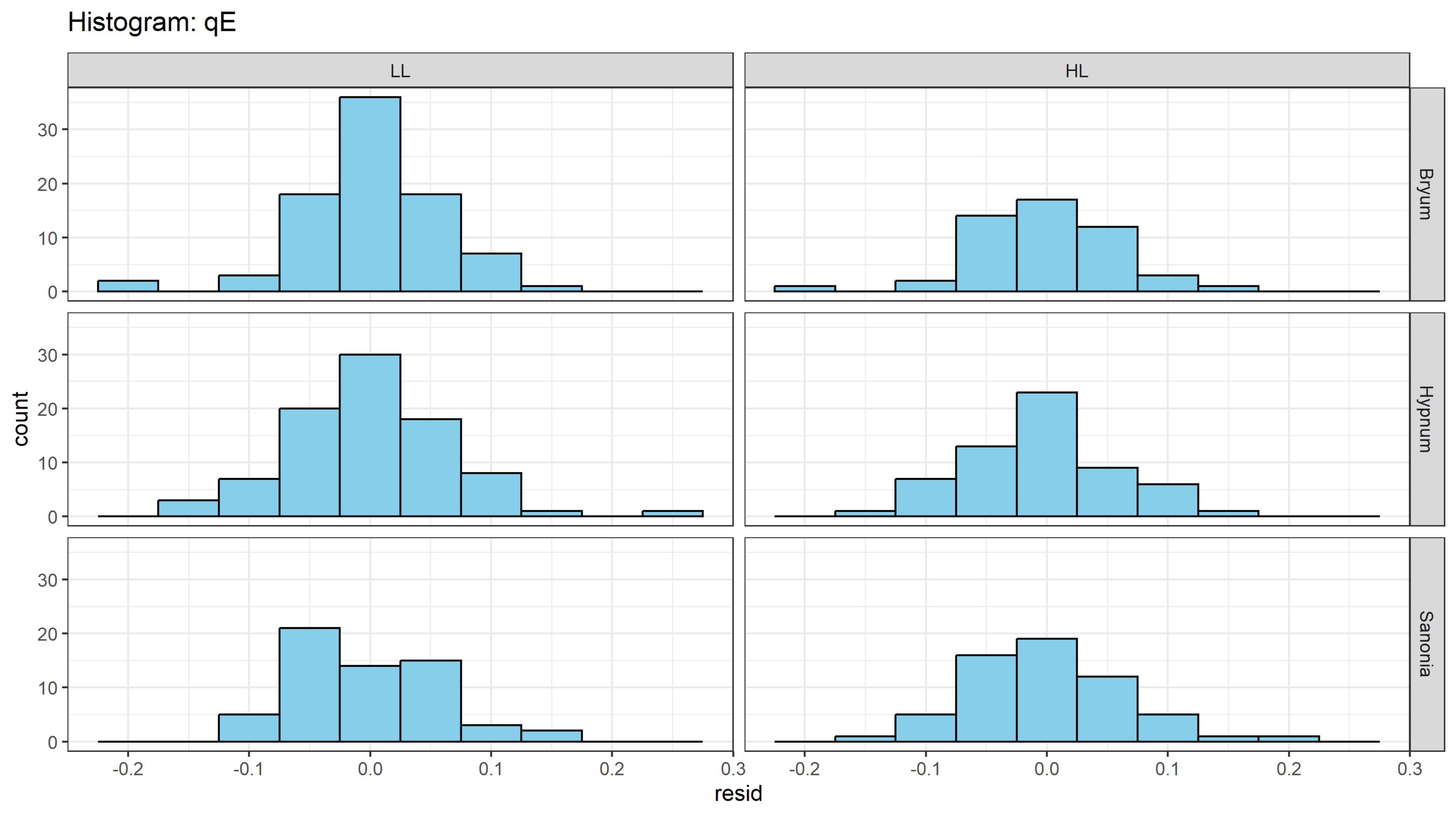
Histogram: qE
LL
HL
Bryum
Hypnum
Sanonia
count
resid
0
10
20
30
-0.2
-0.1
0.0
0.1
0.2
0.3

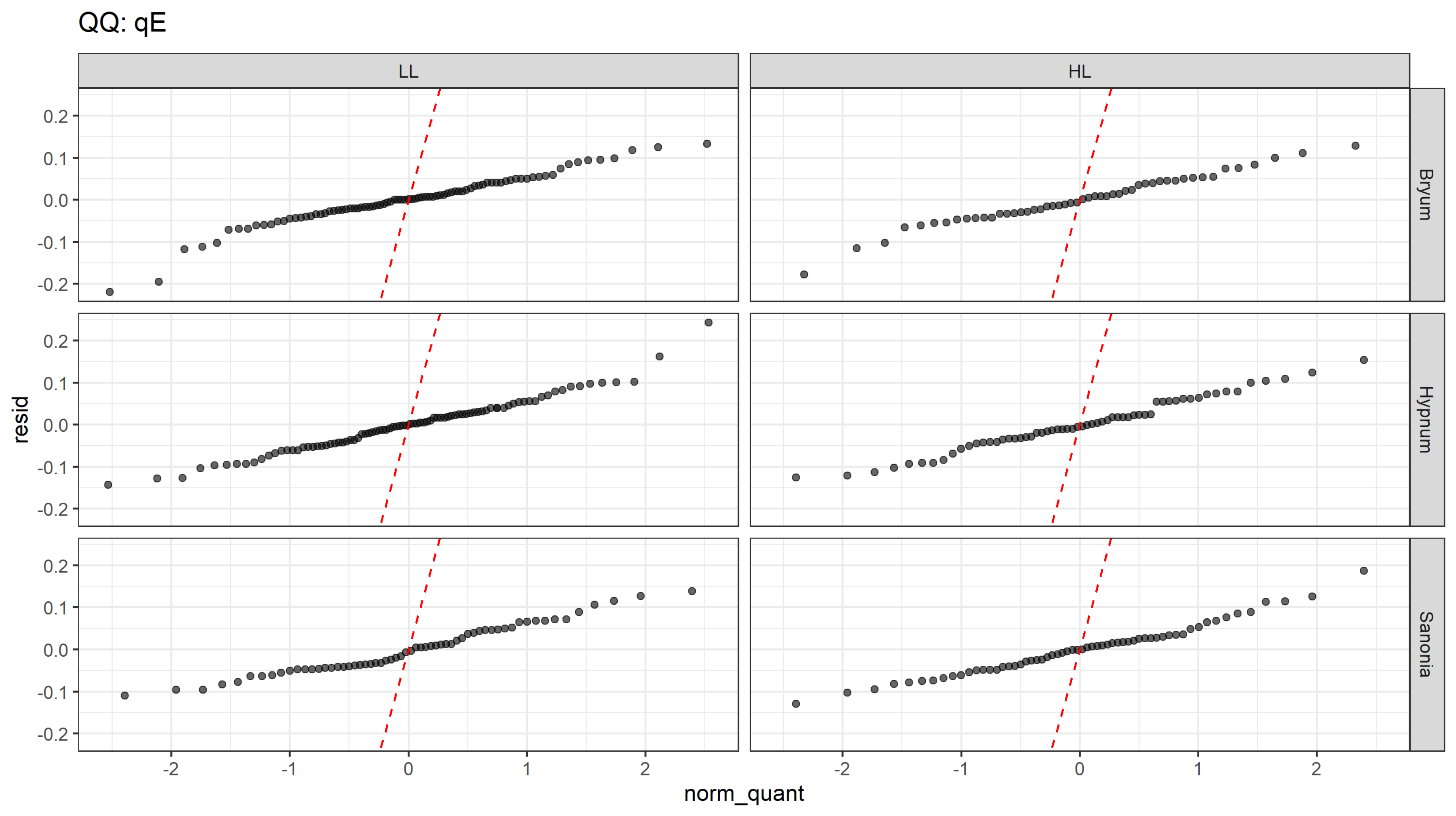
QQ: qE
LL
HL
Bryum
Hypnum
Sanonia
resid
norm_quant
0.2
0.1
0.0
-0.1
-0.2
-2
-1
0
1
2

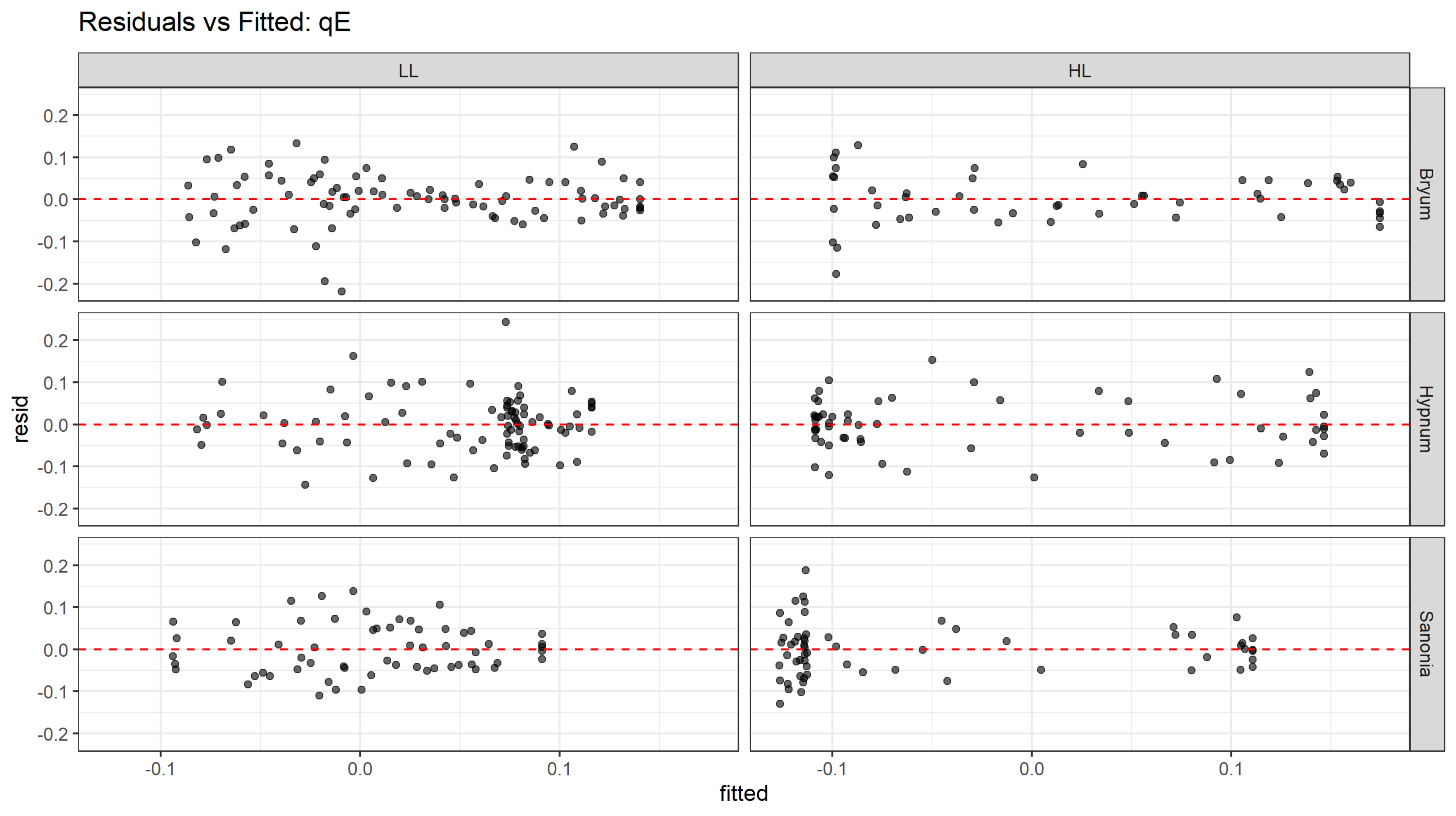

Residuals vs Fitted: qE
LL
HL
Bryum
Hypnum
Sanonia
resid
fitted
0.2
0.1
0.0
-0.1
-0.2
-0.1
0.0
0.1

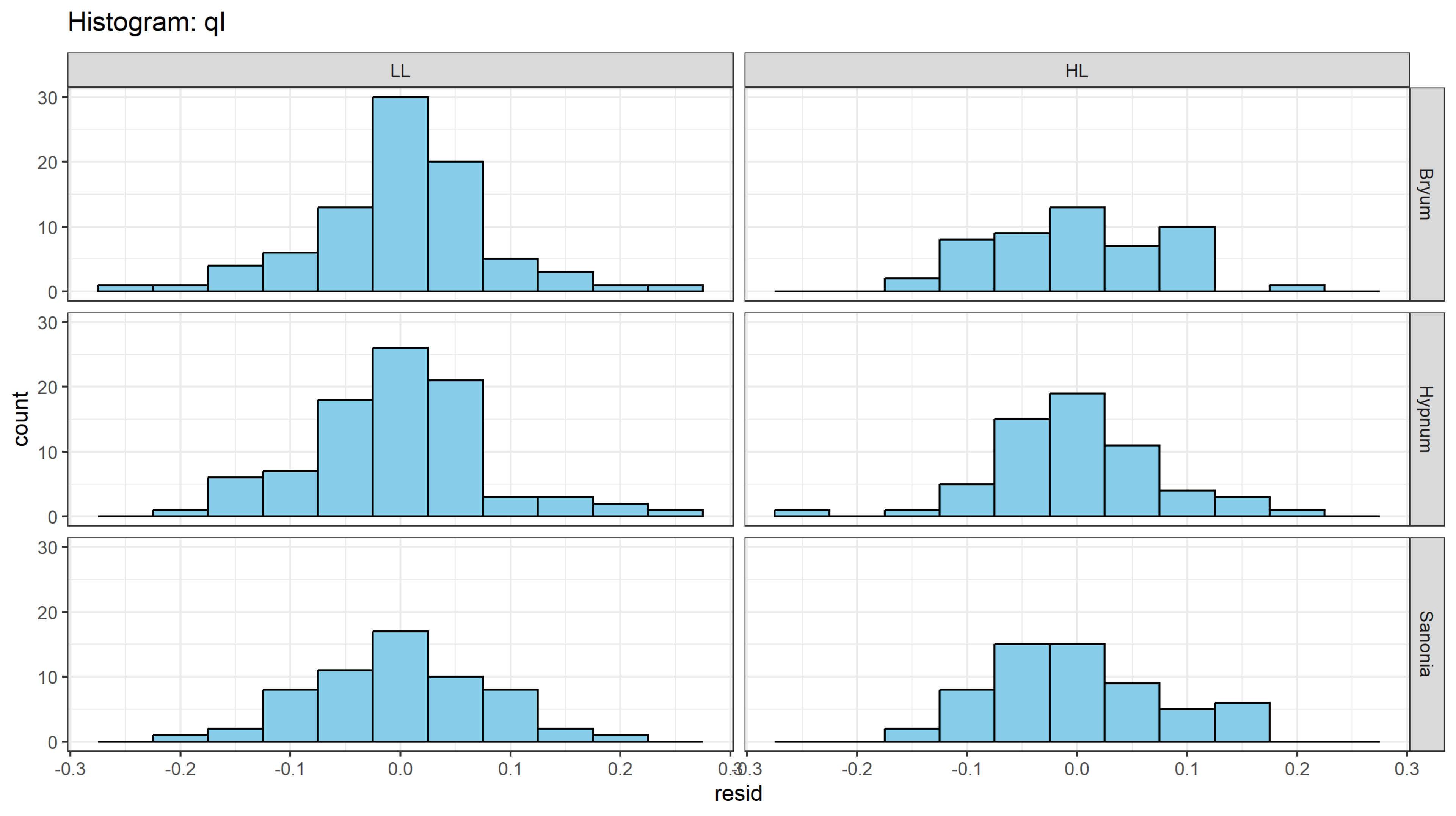

Histogram: ql
LL
HL
Bryum
Hypnum
Sanonia
count
resid
0
10
20
30
-0.3
-0.2
-0.1
0.0
0.1
0.2
0.3

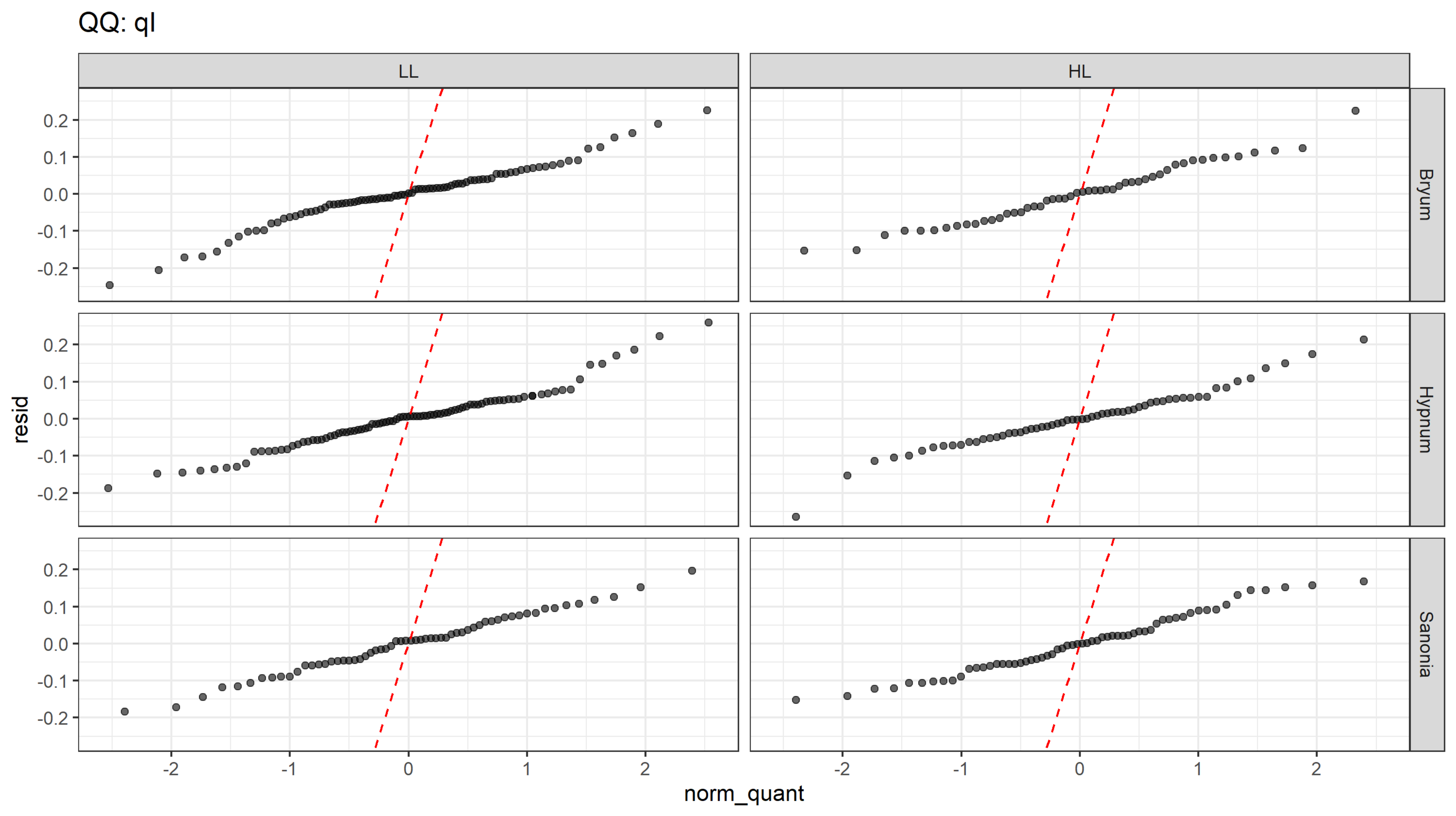

QQ: ql
LL
HL
Bryum
Hypnum
Sanonia
resid
norm_quant
0.2
0.1
0.0
-0.1
-0.2
-2
-1
0
1
2

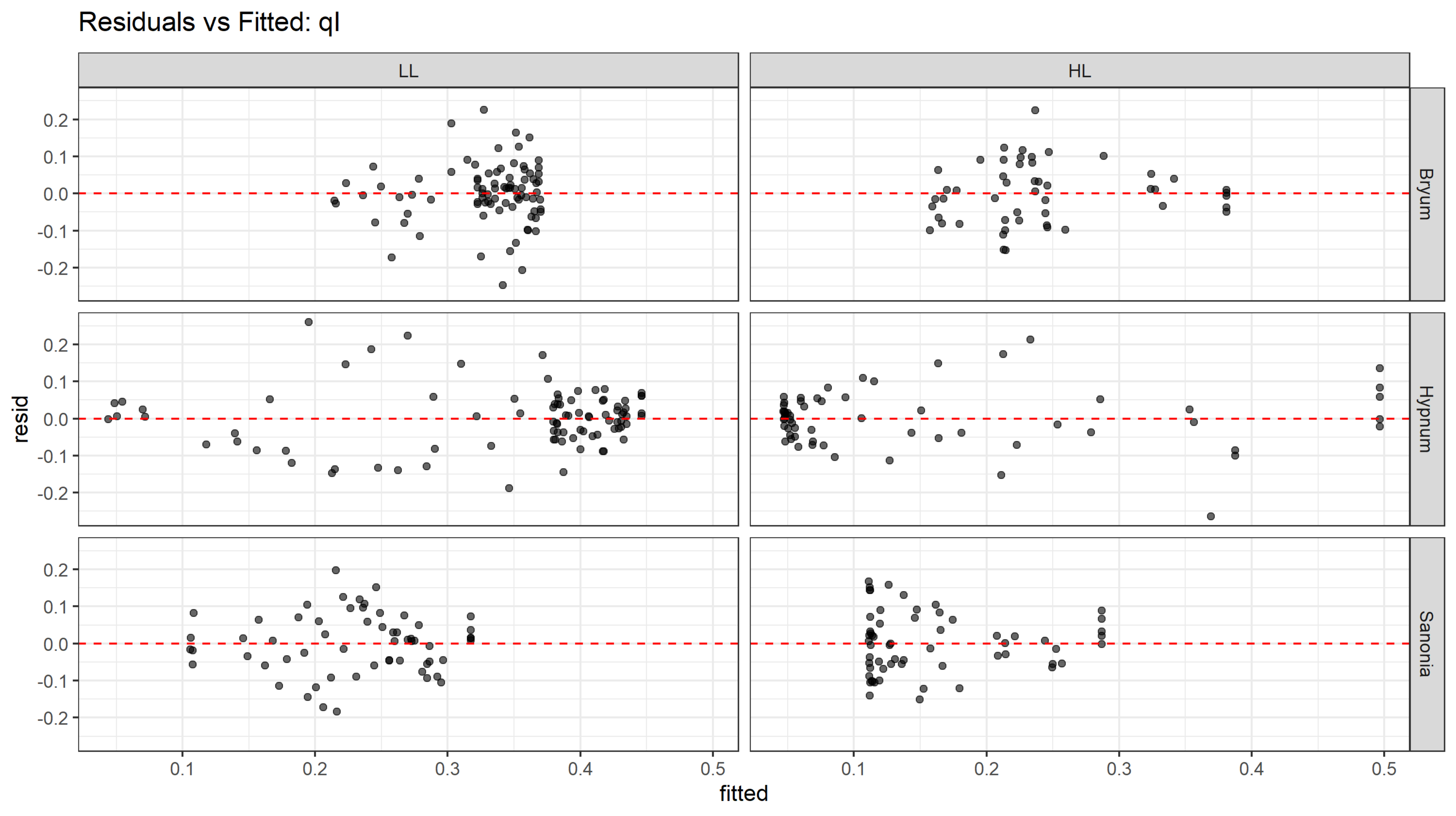

Residuals vs Fitted: ql
LL
HL
Bryum
Hypnum
Sanonia
resid
fitted
0.2
0.1
0.0
-0.1
-0.2
0.1
0.2
0.3
0.4
0.5

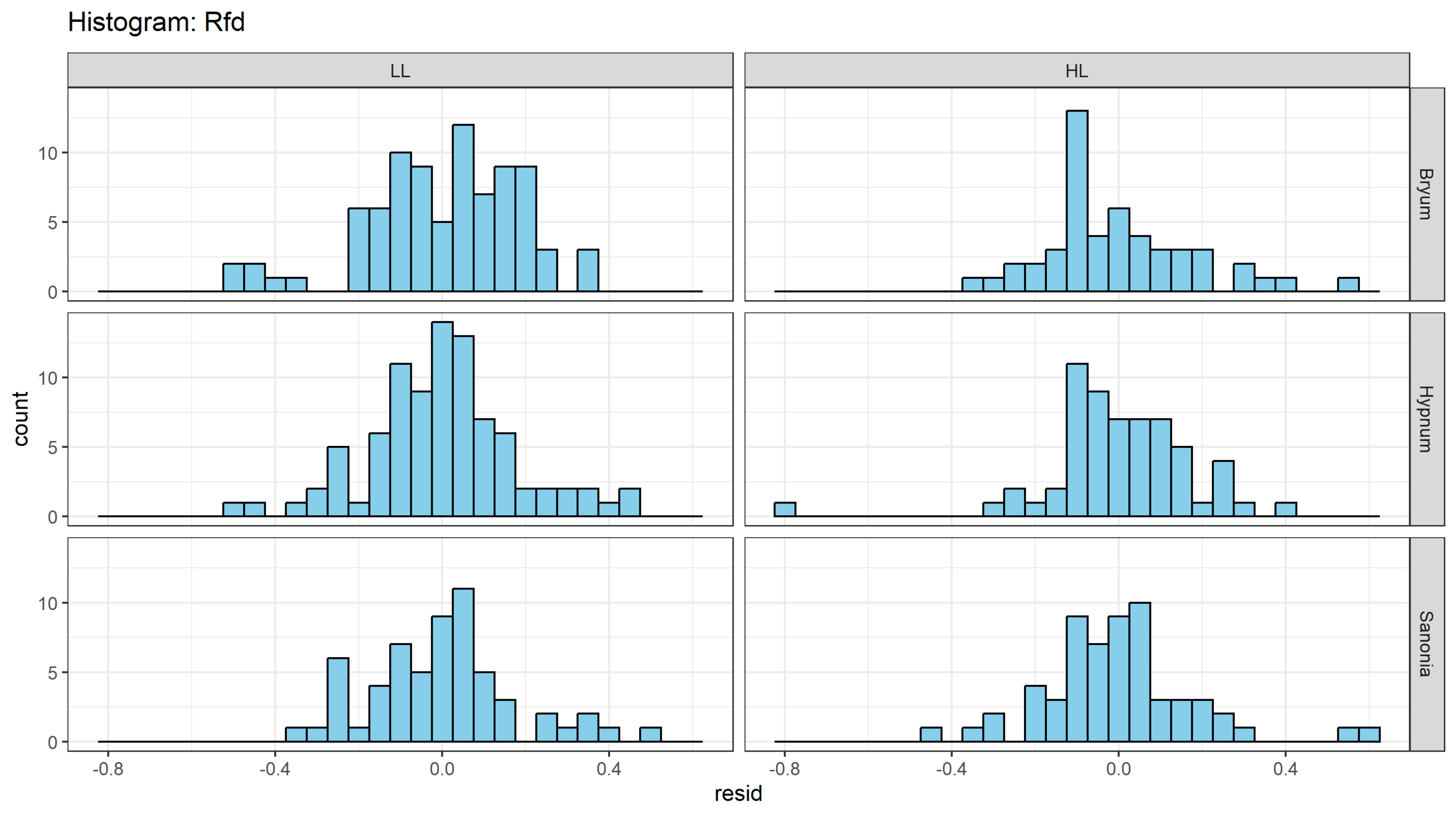
Histogram: Rfd
LL
HL
Bryum
Hypnum
Sanonia
count
resid
0
5
10
-0.8
-0.4
0.0
0.4

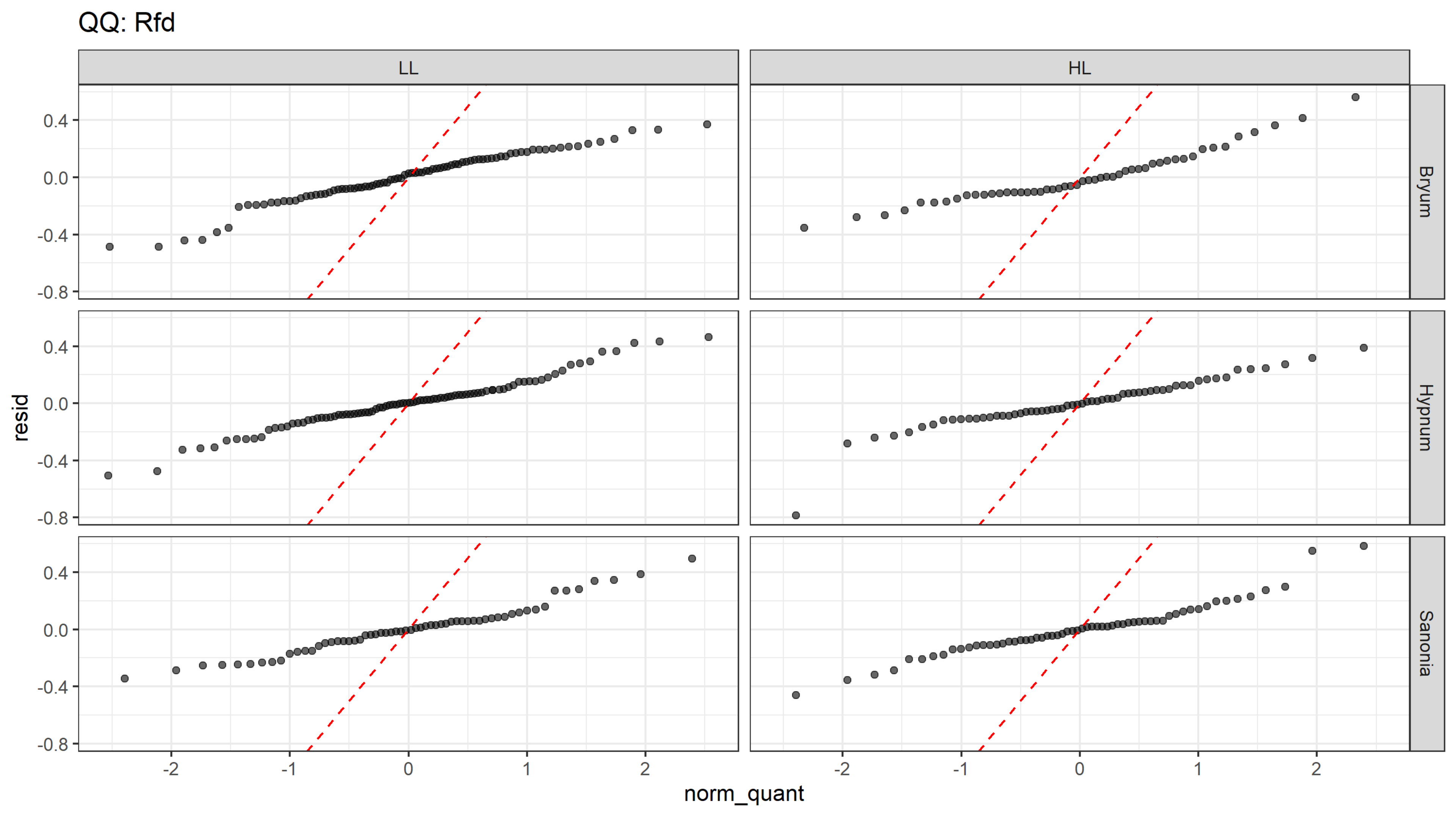
QQ: Rfd
LL
HL
Bryum
Hypnum
Sanonia
resid
norm_quant
0.4
0.0
-0.4
-0.8
-2
-1
0
1
2

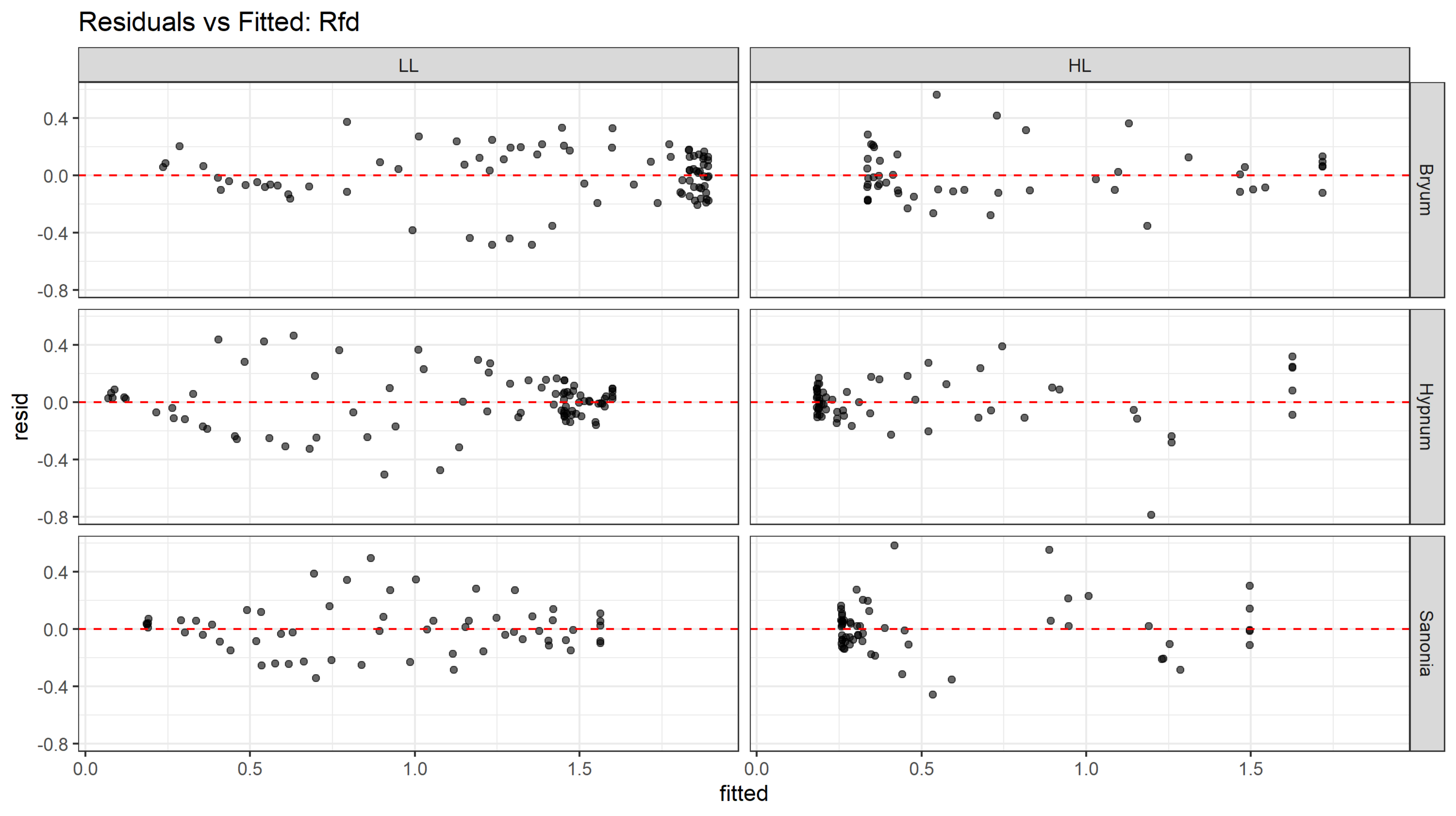

Residuals vs Fitted: Rfd
LL
HL
Bryum
Hypnum
Sanonia
resid
fitted
0.4
0.0
-0.4
-0.8
0.0
0.5
1.0
1.5
