# Supplementary material for: Combination of dehydration and high light exposure effects on PSII photochemistry in three Antarctic moss species
Source: Ann Bot. 2026 Jul 10;138(5):1943–55. doi: 10.1093/aob/mcag207 (PMC13715043; doi:10.1093/aob/mcag207)
Supplement: Supplementary file 1 [file mcag207_supplementary_data.zip › Supplementary Material.docx]

**Supplementary Material**

**Table S1**: parametric coefficients describing the linear effects of Treatment, Species, and their interaction on each ChlF parameter. The intercept (reference level) is set as Bryum under low light (LL). The adjusted R² for each model is reported in the last row as a measure of goodness of fit. Signif. codes: p < 0.001 “***”, p < 0.01 “**”, p < 0.05 “*”, p < 0.1 “.”, p ≥ 0.1 “”

|  | F_M_ | F_0_ | asinh F_M_ | asinh F_0_ |
| --- | --- | --- | --- | --- |
| Intercept | 549.79*** | 190.33*** | 6.777*** | 5.874*** |
| Treatment: HL | -165.73*** | -20.35*** | -0.409*** | -0.14*** |
| Species: Hypnum | 866.73** | 333.29** | 0.936*** | 1.013*** |
| Species: Sanionia | 107.78*** | 77.32*** | 0.048 | 0.284*** |
| Treatment x Species: Hypnum | -375.68*** | -100.13*** | -0.217*** | -0.168*** |
| Treatment x Species: Sanionia | 120.11*** | 27.06* | 0.273*** | 0.115* |
| R^2^ (adj) | 0.932 | 0.92 | 0.957 | 0.927 |

**Table S2**: approximate significance of smooth terms that describes the non-linear effect of RWC on each ChlF parameter within each group (species × treatment combination). Each value corresponds to effective degrees of freedom, edf = 1 indicates a linear relationship, while higher values reflect increasing non-linearity. Signif. codes: p < 0.001 “***”, p < 0.01 “**”, p < 0.05 “*”, p < 0.1 “.”, p ≥ 0.1 “”

| Group | F_M_ | F_0_ | asinh F_M_ | asinh F_0_ |
| --- | --- | --- | --- | --- |
| Bryum LL | 2.577*** | 2.296*** | 2.954*** | 2.536*** |
| Hypnum LL | 3.510*** | 2.696*** | 3.639*** | 2.834*** |
| Sanionia LL | 3.403*** | 3.241*** | 3.729*** | 3.543*** |
| Bryum HL | 3.133*** | 3.237*** | 3.418*** | 3.241*** |
| Hypnum HL | 3.711*** | 3.517*** | 3.500*** | 3.371*** |
| Sanionia HL | 3.476*** | 3.328*** | 3.556*** | 3.305*** |

**Figure S1**: Derivatives functions of smooth curves, for each species and each treatment, of the following parameters: (A) F_V_/F_M_, (B) Φ_PSII_, (C) Rfd, (D) NPQ, (E) qI, (F) qE Blue: LL, red: HL.

**Figure S2**: Derivatives functions of smooth curves, for each species and each treatment, of the following parameters: (A) F_M_, (B) F_0_, (C) asinh_F_M_, (D) asinh_F_0_, (E) asinh_NPQnorm, (F) asinh_qInorm. Blue: LL, red: HL.
